# Generative aptamer discovery using RaptGen

**DOI:** 10.1038/s43588-022-00249-6

**Published:** 2022-06-02

**Authors:** Natsuki Iwano, Tatsuo Adachi, Kazuteru Aoki, Yoshikazu Nakamura, Michiaki Hamada

**Affiliations:** 1 Graduate School of Advanced Science and Engineering, Waseda University, Tokyo, Japan; 2 RIBOMIC, Tokyo, Japan; 3 Computational Bio Big-Data Open Innovation Laboratory (CBBD-OIL), National Institute of Advanced Industrial Science and Technology (AIST), Tokyo, Japan; 4 Graduate School of Medicine, Nippon Medical School, Tokyo, Japan

**Keywords:** Drug discovery, Computational models, Nucleic-acid therapeutics

## Abstract

Nucleic acid aptamers are generated by an in vitro molecular evolution method known as systematic evolution of ligands by exponential enrichment (SELEX). Various candidates are limited by actual sequencing data from an experiment. Here we developed RaptGen, which is a variational autoencoder for in silico aptamer generation. RaptGen exploits a profile hidden Markov model decoder to represent motif sequences effectively. We showed that RaptGen embedded simulation sequence data into low-dimensional latent space on the basis of motif information. We also performed sequence embedding using two independent SELEX datasets. RaptGen successfully generated aptamers from the latent space even though they were not included in high-throughput sequencing. RaptGen could also generate a truncated aptamer with a short learning model. We demonstrated that RaptGen could be applied to activity-guided aptamer generation according to Bayesian optimization. We concluded that a generative method by RaptGen and latent representation are useful for aptamer discovery.

## Abstract (web summary)

A probabilistic generative model for aptamers called RaptGen is introduced, which accelerates the process of aptamer development by generating new aptamer sequences.

## Main

Aptamers are short single-stranded oligonucleotides that bind to specific targets through their three-dimensional folding structure. They are analogous to antibodies and have a variety of applications, including therapeutics^1,2^, biosensors^3^ and diagnostics^4^. The advantages of aptamers are that they are rapidly developed by in vitro generation, are low immunogenic^5^ and have a wide range of binding targets, including metal ions^6^, proteins^7^, transcription factors^8^, viruses^9^, organic molecules^10^ and bacteria^11^. Aptamers are generated by the systematic evolution of ligands by exponential enrichment (SELEX)^12,13^. SELEX involves iterations of affinity-based separation and sequence amplification. This iterative process results in an enriched pool that is analyzed for candidate selection. Recent advances in high-throughput sequencing have enabled us to conduct high-throughput SELEX (HT-SELEX) to collect a vast number of aptamer candidates^14–16^. Current sequencing techniques can evaluate a limited number of reads: approximately 10^6^. Micrograms of a SELEX input library only contains around 10^14^ copies of RNA, whereas an RNA library containing a 30 nt random region theoretically has 10^18^ (~4^30^) unique sequences. Hence we can only evaluate a very small portion of the theoretical diversity, and thus computational approaches that efficiently process high-throughput sequencing data are critical in aptamer development.

Several computational approaches that identify aptamers using HT-SELEX data have been reported. Aptamer identification tools utilize parameters associated with the SELEX principle, such as frequency, enrichment and secondary structure^17–20^. Although they are useful for identifying sequences from HT-SELEX data, various candidates are limited by the actual sequence existence in the data. Simulation-based methods have been reported for sequence generation^21–23^; however, these methods require preceding motif information and are therefore not suitable for identifying aptamers against an unfamiliar target. Computational approaches have also been developed to predict aptamer motifs. Motif prediction is useful not only for candidate discovery but also for aptamer development processes such as truncations and chemical modifications. Several methods have been developed for motif detection by using secondary structures^24^, enrichment of subsequences during SELEX experiments^25^ and emphasis on various loop regions^26^. In addition to these approaches, AptaMut utilizes mutational information from SELEX experiments^22^. As nucleotide substitutions can increase aptamer affinity, mutational information is beneficial for candidate discovery. However, although insertions and deletions are also important factors for altering aptamer activity, in silico methods that deal with these mutations are poorly developed; thus, a method that generates sequences from experimental data is needed to expand the exploratory space, and including motif information and nucleotide mutations confer an increased opportunity for aptamer discovery.

We focused on a neural network to develop a procedure for aptamer generation and motif finding. As reported previously, neural networks are suitable for analyzing large datasets and are compatible with high-throughput sequencing data. DeepBind adopts a convolutional neural network (CNN) to distinguish DNA motifs from transcription factors and find sequence motifs by visualizing network parameters^27^. Recurrent neural networks can also be used for sequence discovery^28,29^. Neural network-driven generative models are currently being applied in a broad range of research areas. Some examples of neural network-dependent generative models include deep belief networks^30^, variational autoencoders (VAEs)^31^, and generative adversarial networks^32^. For a probabilistic generation of nucleic sequences, using long short-term memory (LSTM) was proposed to mimic sequence distribution^33^. Generative adversarial network-based sequence generation methods have also been proposed^34^.

Variational autoencoder-based compound designs have been reported in small molecule discovery. VAEs learn a representation of the data by reconstructing the input data from a compressed vector^31^. Kusner and colleagues used grammar-based VAEs and SMILES sequences to generate chemical structures for activity optimization^35^, and Gómez-Bombarelli et al. used the representation learned by the VAE to design chemical compounds^36^. Unlike other generative models, VAEs exploit the relationship between compressed feature space and inputs in a bidirectional manner; they are therefore suitable for visualizing similarity-oriented classifications and emphasizing important sequence features. Using VAEs to convert HT-SELEX data into low-dimensional space would be useful for candidate discovery; thus, VAE-based aptamer generation systems are worth investigating. When conducting VAE modeling for HT-SELEX data, having a profile hidden Markov model (HMM) decoder should be beneficial for aptamer discovery; it captures motif subsequences—robust with substitutions, deletions and insertions—and can easily monitor effects from the subsequences.

Here we present RaptGen, a VAE for aptamer generation. RaptGen uses a profile HMM decoder to efficiently create latent space in which sequences form clusters based on motif structure. Using the latent representation, we generated aptamers not included in the high-throughput sequencing data. Strategies for sequence truncation and activity-guided aptamer generation are also proposed.

## Results

### Overview of RaptGen and its applications

**Fig. 1:**
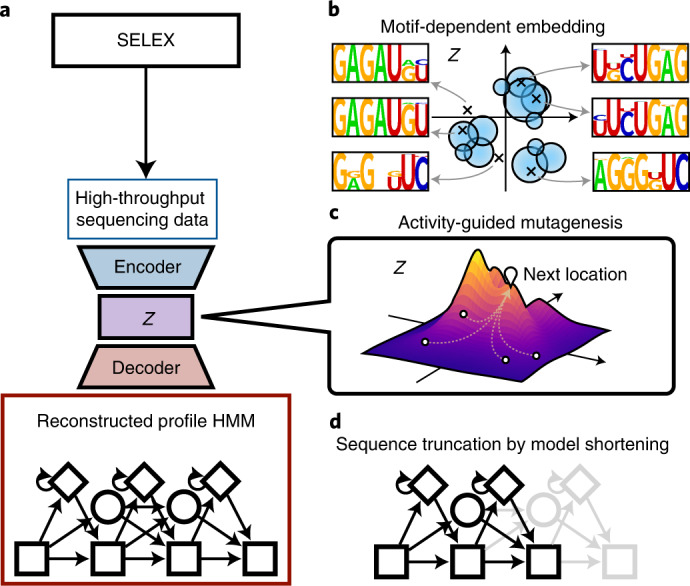
Overall RaptGen schematic and its applications. **a**, RaptGen workflow. RaptGen is a VAE with a profile HMM for decoder distribution, which considers insertions and deletions. Through training, RaptGen learns the relationship between HT-SELEX sequencing data and latent space embeddings (the latent space is shown in *Z* in this figure). **b**, RaptGen constructs a latent space based on sequence similarity. It can also generate intermediate representations with no training data. **c**, RaptGen can propose candidates according to the activity distribution by transforming a latent representation into a probabilistic model. **d**, RaptGen can perform in silico sequence truncation using a short-profile HMM decoder.

RaptGen is a probabilistic generative model that enables us to generate new aptamer sequences that are not included in the input SELEX dataset. To realize this, RaptGen employs a VAE with a profile HMM for decoder distribution and embeds RNA sequences from the input dataset into low-dimensional latent space (Fig. 1a). Using a profile HMM for the decoder renders RaptGen more robust for substitutions and indels in RNA aptamers, thereby achieving better generative performance than existing models (see the ‘Motif-dependent embeddings using simulation data’ section). See Methods for details on RaptGen’s procedures.

In this study we propose three important applications of RaptGen for aptamer discovery. First, a latent space learned by RaptGen is visualized with a sequence motif, and new aptamer sequences are generated for an arbitrary point in the latent space (see Fig. 1b and the ‘Real data evaluation with RaptGen’ section). Second, optimized aptamer sequences are searched in the latent space by considering additional experimental information, such as the binding affinity of a subset of sequences (see Fig. 1c and the ‘RaptGen application in aptamer discovery’ section). Third, RaptGen enables in silico the design of truncated aptamer sequences using a shorter-profile HMM decoder (see Fig. 1d and the ‘RaptGen application in aptamer discovery’ section).

### Motif-dependent embeddings using simulation data

**Fig. 2:**
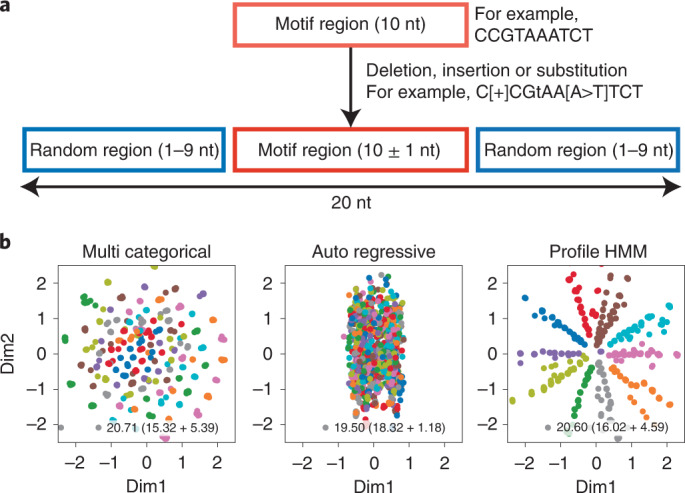
Results for simulated data. **a**, Scheme of simulation data used for evaluating the decoder models. Ten different motifs with a 10% chance of having nucleotide mutations were randomly extended to 20 bases. **b**, Embeddings of hypothetical motifs using different decoder models. The simulation data obtained in **a** were subjected to the VAE with the multicategorical, autoregressive and profile HMM. The resulting latent space is shown, where Dim1 and Dim2 are the first and second axis in the space, respectively. The ELBO is in the right bottom corner with the reconstructed error and Kullback–Leibler divergence. Each motif is plotted with different colors. Source data

We first attempted to construct a VAE with an encoder and decoder applicable to aptamer discovery. In the aptamer representation space, sequences containing the same motif should be in a neighboring area. Robustness against nucleotide mutations and motif positions should also be considered. We investigated different types of sequence representation models to identify a desirable decoder. We constructed VAEs with a CNN encoder and three different types of probabilistic models (the multicategorical model, autoregressive model and profile HMM) as a decoder. Simulation data, including ten different motifs, were created to assess the visualizing capability of these VAEs (Fig. 2a). We observed that profile HMM-embedded sequences in a motif-dependent manner after training the data, whereas the multicategorical and autoregressive models displayed indistinctive distributions (Fig. 2b). The evidence lower bound (ELBO) was calculated to evaluate the model. Although the multicategorical model and profile HMM had almost the same ELBO (20.71 and 20.60), and had similar reconstitution errors (15.32 and 16.02) and Kullback–Leibler divergence scores (5.39 and 4.59), the embedding space of the multicategorical model failed to visualize a motif cluster. This is thought to be due to the inability of the multicategorical model to consider motif positions. As the nucleotide probability of each position was independently estimated in the multicategorical model, the same motifs in the shifted position might not be aligned in latent space. The autoregressive model had the lowest ELBO (19.50); however, the reconstitution error was the worst (18.32). Furthermore, the classification result was not optimal. We suppose that latent representation is dispensable in the autoregressive model as the model itself has context information. We also compared the different encoder types. Long short-term memory^37^ and CNN–LSTM were evaluated in combination with the above three decoders. Long short-term memory is used in character-level text modeling. The embedding space from the multicategorical and autoregressive models was still inadequate using either encoder (Supplementary Section 8). Profile HMM created distinguishable embedding with LSTM, whereas a learning deficiency was observed in combination with CNN–LSTM (Supplementary Section 8). Collectively, we concluded that the profile HMM decoder is favorable for motif-dependent embedding. A VAE composed of a CNN encoder and a profile HMM decoder was examined in the following study.

**Fig. 3:**
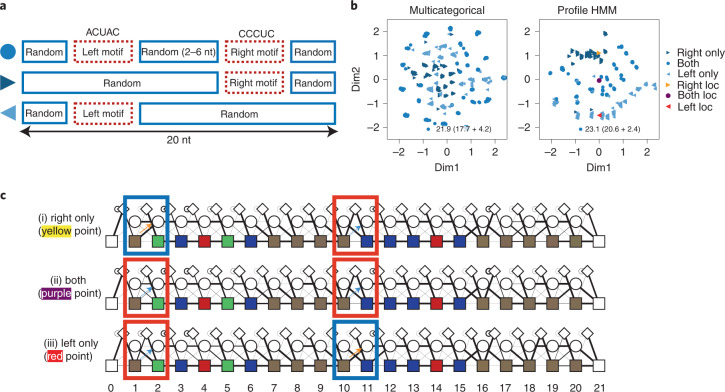
Results for simulated data with complex motif. **a**, Scheme for paired motifs with simulation data. A set of 5 nt was used with two- to six-base insertions and extended to 20 bases. Data containing one motif were also generated. **b**, Embeddings of split motifs by the VAE. Simulation data generated in **a** were analyzed using the profile HMM VAE. The resulting embedding plot is shown. Plots generated by the VAE with the multicategorical model decoder are also shown for comparison. **c**, Representative profile HMM was obtained from the profile HMM VAE. The profile HMM indicated in **b** is shown. The thickness of the line represents the transition probability. The color of the matching state (filled rectangle) indicates the probability of emitting each nucleotide. A, U, G and C are green, red, yellow and blue, respectively; brown shows the state emitting A, U, G and C equally. The blue rectangles show a high probability of skipping the motif by moving to the deletion state, whereas the red rectangles highlight a high probability of including the motif. Source data

We next tested whether our VAE model could distinguish split motifs. Subsequence co-occurrence at distances is often observed in RNA due to intramolecular base-pairing and internal-loop structures^38^. We applied simulation data with a pair of 5 nt split motifs to the VAE (Fig. 3). The multicategorical model decoder was used for comparison. Figure 3b shows the results of embedding split motifs. Plots are displayed in three groups: right motif-, left motif- and both motif-remaining sequences. Profile HMM output sequences related to the motif, whereas the multicategorical model scattered the sequences. We sampled representative profile HMM distributions from each population. Profile HMM visualization shows that the yellow point skips the left motif. The red point skips the right motif, both by allocating a high probability of jumping to the deletion state from the matching state (Fig. 3c). Visualization of the purple point shows that the middle of two points has a low probability of skipping either of the motif fragments. The transition probability to skip the left motif (${a}_{{M}_{1},{D}_{2}}$) and the right motif (${a}_{{M}_{10},{D}_{11}}$) for right-only-, both- and left-only-motif models was (0.995, 0), (0.107, 0.002) and (0, 0.987), respectively. Interestingly, the point located between these two motifs has a high probability of including both motifs. These results show that a profile HMM decoder is also applicable for split motifs. Hereafter, we called a VAE with a profile HMM decoder RaptGen.

### Real data evaluation with RaptGen

We further evaluated RaptGen using SELEX sequence data obtained from our previous study^20^. As real data are more complex than simulation data, we first investigated the dimensions of the latent space. Raw HT-SELEX data have 30 or 40 nt variable regions and fixed primer regions at both ends. In the present study, we used the variable region to create latent space. We tested up to twelve spatial dimensions and trained the model 50 times on datasets A and B (Supplementary Fig. 1). For Dataset A, the minimum loss was in four dimensions, and the second-lowest was in two dimensions. For Dataset B, the minimum loss was in three dimensions and the second-lowest was in two dimensions. Loss tended to increase as the embedding dimension increased; however, the loss of one-dimensional space was higher than that of the ten-dimensional space. The lower dimension would be favorable for visualization, and performing Bayesian optimization would be advantageous, as described in later sections. We therefore adopted a two-dimensional space for analysis.

**Fig. 4:**
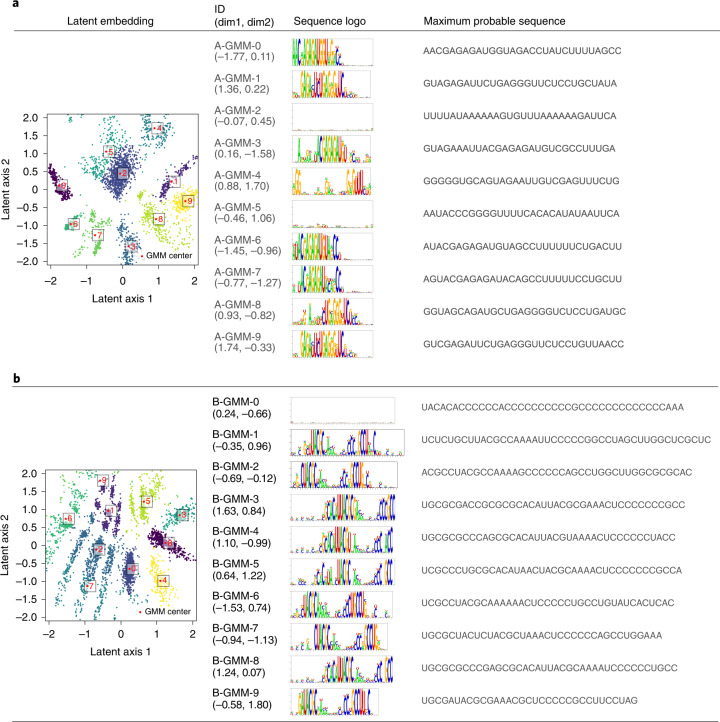
RaptGen applications for real data. The latent embeddings and reconstituted sequences through GMM. **a**,**b**, Sequences of datasets A (**a**) and B (**b**) were analyzed by RaptGen. The latent embeddings of datasets A and B are shown in the first column, where clusters were estimated by GMM. The plot colors indicate the different clusters. Profile HMMs were obtained from each center of the Gaussian distributions. Their ID and locations in latent space, and logo views of profile HMM (compare with Supplementary Section 1) are listed in the second and third columns, respectively. Sequences for activity evaluation were reconstituted from each profile HMM. The maximum probable sequences were listed in the fourth column. Source data

We next subjected two independent HT-SELEX datasets (datasets A and B) to RaptGen. The resulting latent embeddings are shown in Fig. 4 and Supplementary Section 4. We previously demonstrated that aptamers from datasets A and B exhibit continuous and split motifs, respectively. As the SELEX experiment sequences are amplified with specific binding motifs, we reasoned that they would form clusters in a latent space based on their motifs. We thus used the Gaussian mixture model (GMM), which hypothesizes that data consists of a mixture of Gaussian distributions, to classify the distributions. We chose ten different points representing the latent cluster center of the GMM (Fig. 4). We observed that sequences with an uncertain profile HMM such as A-GMM-2, A-GMM5 and B-GMM-0 were embedded near the latent space center. Unenriched aptamer sequences remained after the SELEX experiments. We predicted that these junk sequences formed clusters in the latent space. By contrast, the near-edge area contained sequences that emit nucleotides preferentially. We also confirmed that similar profiles were embedded in similar areas (Supplementary Section 4). These results provide support for the use of RaptGen to analyze high-throughput SELEX data.

We attempted to generate the most probable sequence from the profile HMM of each GMM center for activity evaluation. We calculated the model state path with the highest probability and derived the most probable sequence according to the path. When the path included insertion states, we generated up to 256 sequences with no duplication by randomly replacing each insertion state with a single nucleotide and selected a sequence with the highest probability. The resulting reconstituted sequences and their probabilities are shown in Fig. 4. After connecting with their fixed primer sequences, aptamer RNAs were produced by in vitro transcription and their binding activities were assessed by surface plasmon resonance assay. Aptamers identified in our previous study were used as positive controls^20^. Although more than half of the candidates were found to have weak or no activity, some sequences such as A-GMM-1, B-GMM-4 and B-GMM-8 had evident binding activity. To determine whether these aptamers exist in the original data, we calculated each sequence’s edit distance from the nearest HT-SELEX sequence (Supplementary Table 1). It should be noted that all candidate sequences were not included in the original SELEX data. Collectively, we concluded that RaptGen enables us to generate aptamers from the latent space and reduces the limitations of working with actual sequence data.

### RaptGen application in aptamer discovery

**Fig. 5:**
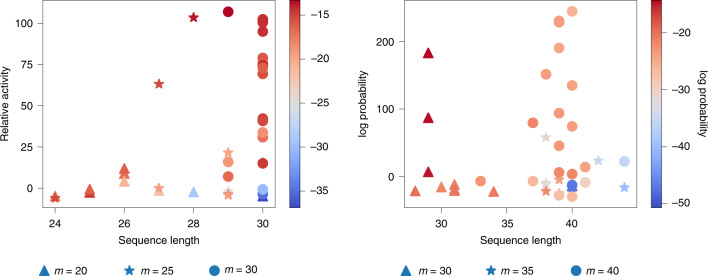
Truncated aptamers obtained from RaptGen. A profile HMM decoder with a shorter model length, *m*. A 20 or 25 nt decoder was used to analyze Dataset A, in which the random region of the SELEX library is 30 nt. Similarly, a 30 nt or 35 nt decoder was used to analyze Dataset B, in which the random region of the SELEX library is 40 nt. Ten candidate profile HMMs were newly obtained by GMM. After reconstitution of the maximum probable sequences, aptamer activities were assessed by surface plasmon resonance. Scatter plots of the relative activities of aptamers and their lengths are shown, including aptamers tested in Fig. 4. Different markers indicate different lengths of the profile HMM decoder. Colors indicate the log probability of a sequence. Source data

We proposed further applications of RaptGen for aptamer development. Shortening the aptamer length is important for industrial application. Aptamer truncation can reduce the cost of manufacturing and facilitate material quality assurance. It also prevents unexpected biochemical interactions. Hence, aptamers should be shortened as much as possible. As the profile HMM can handle variable sequence lengths, learning settings could diverge from the original SELEX library. For example, a decoder model does not require the same length of the random region. We attempted to generate shorter aptamers than SELEX with RaptGen. We introduced a short-profile HMM with truncated length by 5 or 10 nt from the original SELEX design. Dataset A was analyzed with a 20 nt and 25 nt model (called A-L20 and A-L25), where the initial library was 30 nt. Dataset B was analyzed with a 30 nt and 35 nt model (called B-L30 and B-L35), where the initial library was 40 nt. After creating latent space, ten sequences for each length were created in a GMM-dependent manner described above. Figure 5 shows the relative activity of proposed aptamers with their lengths. For Dataset A, the 28 nt candidate showed binding activity where the initial library was 30 nt. For Dataset B, the 29 nt candidate showed considerable activity compared with the original setting, which was 40 nt. These results suggest that RaptGen can generate a shorter aptamer than the experimentally expected length. We found that sequences with low reconstitution probability tended to have low binding activity and that sequences showing binding activity had relatively high probability (Fig. 5). This observation would be helpful for effective candidate selection. We observed a tendency of sequence extension in datasets A-L20, A-L25 and B-L35. For instance, in Dataset A, 26 nt sequences were generated from the 20 nt RaptGen setting. We speculate that the profile HMM is prone to imitating the original length in some situations. The optimal truncation length was different for each dataset. We did not identify the cause of this difference. Further studies should be performed to determine efficient truncation.

**Fig. 6:**
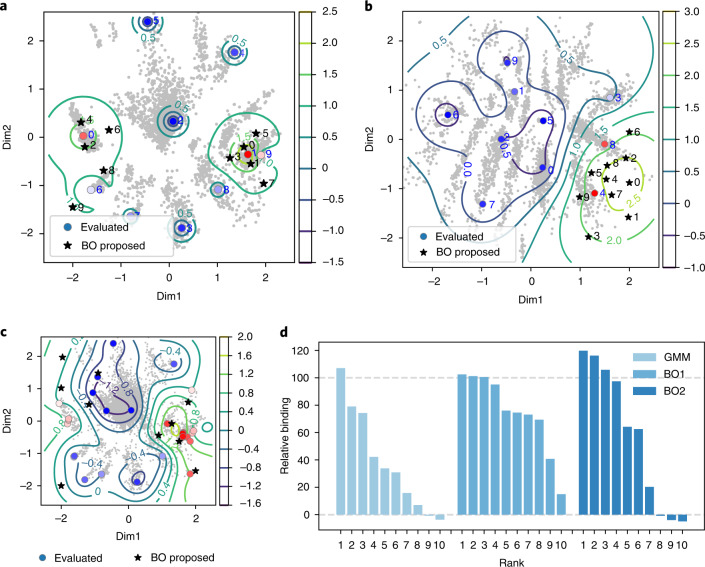
Results of Bayesian optimization for real data. **a**,**b**, The activity distribution and proposed Bayesian optimization (BO) points for datasets A (**a**) and B (**b**). Binding activity data shown in Fig. 4 were embedded into latent space. Gray points indicate latent embeddings shown in Fig. 4. The contour line overlaid on the embeddings indicates the predicted activity level. This is the acquisition function of Bayesian optimization, which is the upper confidence bound of the posterior distribution of the Gaussian process (GP-UCB)^52^. Ten points were proposed by the Bayesian optimization process with a local penalization function. Circles represent the re-embedded position of the GMM centers. Red and blue indicate high and low binding activity, respectively. Stars represent the locations proposed by Bayesian optimization. **c**, The embedding space and the next value to be proposed in it. The evaluated sequences are color-coded according to their sequence binding affinities. Black stars represent the next ten proposed points resulting from Bayesian optimization. **d**, Relative affinities of sequences proposed by different methods; BO1 and BO2 indicate the iterations of Bayesian optimization performed. Rank indicates the within-method activity ranking. Source data

In another application of RaptGen, we generated aptamers using activity information. Aptamer derivatives harboring nucleotide mutations should be distributed around the mother sequence in the latent space. To predict effective candidates from the neighboring area of an active aptamer, binding activity distribution should be predicted. We used a Bayesian optimization algorithm for learning an activity distribution. As the distribution for the Bayesian optimization process is required to be of low dimension, RaptGen is suitable for this strategy. To implement Bayesian optimization, we first embedded activity data in the latent space. The sequences listed in Fig. 4 were reconverted into the space. Several locations moved from the initial GMM center (Fig. 6a,b). We used these re-embedded positions to perform Bayesian optimization. The resulting predicted activity distributions are shown in Fig. 6a,b. We used the local penalization function to propose multiple candidates in parallel^39^. Ten profile HMMs were proposed and evaluated for their activity. As shown in Fig. 6a,b, candidates were generated from the peripheral area of the positive clone. We confirmed that new aptamers incorporated nucleotide substitutions (Fig. 4). In addition, most of them had binding activity. Similar results were obtained for both datasets A and B. We further tested the hypothesis that repeated Bayesian optimization could support the generation of superior aptamers. We conducted an additional Bayesian optimization round (BO2) against Dataset A. After generating ten new candidates, we obtained an aptamer with approximately 20% greater binding activity (Supplementary Table 2 and Fig. 6c,d). These results indicate that RaptGen can propose aptamer derivatives in an activity-guided manner and can provide opportunities to optimize their activities.

The present version of RaptGen does not consider the secondary structure of aptamers. Secondary structure information is critical for identifying active aptamers^19,20^. In this subsection, we performed RNA secondary structure analyses for the aptamers obtained in the previous sections. Here we utilized the Rtools webserver^40^ for secondary structure analyses; the results are shown in Supplementary Fig. 3 (Dataset A), Supplementary Fig. 4 (Dataset B) and Supplementary Data 1 (more detailed results for datasets A and B). For Dataset A, the root of the structures (that is, structures around 3′-part) gradually changed according to the relative activity (Supplementary Fig. 3), whereas the stem-loop region around the middle of the sequence was conserved. This result indicates that our Bayesian optimization process optimizes the root of the structures to increase binding abilities. For Dataset B, we observed that high-affinity aptamers tended to form a specific structure (for example, B-GMM-4, B-BO-4 and B-BO-5) compared with the positive control (Supplementary Fig. 4). To confirm the reliability of this analyses, we also performed RNA secondary structure analyses using RNAfold^41^ (Supplementary Figs. 5 and 6 for datasets A and B, respectively), where a consistent result was obtained for Dataset A. For Dataset B, both tools showed high-affinity aptamers tend to form similar structures (note that both tools also suggest that secondary structures in Dataset B are unstable, that is, including relatively low base-pairing probabilities in predicted structures).

## Discussion

One of the popular models for handling high-throughput sequencing data (such as the HT-SELEX data this study focused on) is a discriminative model that distinguishes real aptamer sequences from non-aptamer sequences; examples include DeepBind^27^ and DeeperBind^28^. In training the discriminative models, both positive and negative sequences are necessary; positive sequences are usually shuffled to provide the negative data. As discriminative models are specialized for classification, they cannot generate new sequences. Conversely, RaptGen is a generative model that does not require negative data for training and can generate new aptamer sequences not included in the input sequences. This feature is essential for the three applications (see Fig. 1b–d) investigated in this study. Note that we compared RaptGen with DeepBind regarding motif detection performance (Supplementary Section 9). Jinho et al. proposed LSTM-based sequence generation using SELEX data^33^. However, they did not consider sequence cluster information. RaptGen embeds sequences into a feature representation space, and thus RaptGen could visualize sequence classification and generate representative sequences from each cluster (Fig. 1). Moreover, this low-dimensional representation enables us to conduct Bayesian optimization, which is beneficial for generating variant sequences (Fig. 4). Hence, we believe that RaptGen is a superior generative model compared to LSTM. Hoinka et al. introduced several tools for aptamer discovery, such as AptaCluster, AptaMut and AptaSim^22^. AptaCluster and AptaMut consider mutation information that is derived from the base substitution error rate of the polymerase enzyme. AptaCluster evaluates only actual sequencing data, whereas RaptGen can generate sequences that are not included in the sequencing data. We confirmed that the generated sequences did not appear in the SELEX experiment (see Supplementary Table 1, in which positive edit distances indicate that the corresponding sequence was not included in the original SELEX data). In addition, AptaMut deals with base substitutions but not insertions or deletions. As RaptGen has a profile HMM, it can embed indel information. This capability was confirmed using simulation data (Fig. 2). Because of indel tolerance, RaptGen could also generate sequences shorter than the actual sequencing data (Fig. 5), whereas AptaMut does not estimate such virtual sequences. We therefore believe that RaptGen incorporates mutational information better than AptaMut. In summary, to the best of our knowledge, there are no other data-driven methods to design optimized and truncated aptamers directly from HT-SELEX data, and we believe that RaptGen will be a key tool for efficient aptamer discovery.

In this study, we demonstrated that RaptGen could propose candidates according to activity distribution. According to Bayesian optimization, a sequential construction of posterior distribution would allow us to optimize activity in the latent space. For another instance of Bayesian optimization application, one could set the acquisition function to various indicators other than the binding activity. We could therefore generate candidates according to other properties of interest, including inhibitory activity against enzymes or protein–protein interactions. The application of RaptGen for this purpose is promising.

Although RaptGen helps visualize and understand sequence motifs, this method has computational cost due to sequence probability calculation. Compared with the multicategorical model, which can calculate the sequence independently by position, and the autoregressive model, which only needs calculation on the previous nucleotides, profile HMM requires calculation on all possible state paths and previous (sub)sequences. The offset calculation cost for multicategorical, autoregression and profile HMM is ${{{\mathcal{O}}}}(1)$, ${{{\mathcal{O}}}}(l)$ and ${{{\mathcal{O}}}}(lm)$, respectively, where *l* is the number of previous characters including itself, and *m* is the model length of the profile HMM. Profile HMM also needs to frequently calculate the costly logsumexp function, leading to a longer training time. Additional studies are necessary to improve these issues.

There are two possible extensions of RaptGen. First, as shown in the previous section (see the ‘RaptGen application in aptamer discovery’ section), including the secondary structure in the sequence probabilistic model would improve RaptGen performance. In this direction, an alternative model such as profile stochastic context-free grammar^42^ will be tested in follow-up studies. Another direction of the extension of RaptGen is to consider RNA sequences in all rounds in HT-SELEX experiments.

RaptGen could advance HT-SELEX data-driven RNA aptamer generation. As an RNA aptamer binds to the target protein by the structural complementarity, not by hybridization, interaction between the RNA and the protein is hardly predicted without binding experiments such as SELEX. Once enough number of aptamer–protein pairs and binding data is accumulated, de novo aptamer design without wetlab experiments will be realized in the future. Additionally, simulation-based methods such as molecular dynamics will also be effective to improve computational aptamer design (for example, optimization of aptamers^43^).

## Methods

### Overall study parameters

The VAE proposed in this study is a CNN-based encoder with skip connections and a profile HMM decoder with several training methods. Two simulation datasets containing different types of motifs were generated to assess the interpretability of the decoder. Two independent HT-SELEX datasets were subjected to the VAE, and the GMM was used for multiple candidate selection. Furthermore, Bayesian optimization was performed based on the activities of tested sequences proposed by GMM, and sequences were truncated by shortening the model length. The process is explained in detail in the following sections. An overview is shown in Fig. 1.

### Architecture of the RaptGen model

#### VAE

Variational autoencoders consist of an encoder neural network that transforms input sequence **x** into latent distribution *q*_*ϕ*_(**z**∣**x**) and a decoder neural network that reconstructs the input data from latent representation **z** by learning *p*_*θ*_(**x**∣**z**) where φ and θ are model parameters. As VAE is a generative model, it can be evaluated by model evidence. However, given a dataset ${\bf{X}}=\{{\bf{x}}^{(i)}\}_{i = 1}^{N}$, the model evidence ${p}_{{{{\mathbf{\uptheta }}}}}\left({{{\bf{X}}}}\right)$ is not computationally tractable. Alternatively, we can maximize the ELBO, ${{{\mathcal{L}}}}({{{\mathbf{\uptheta }}}},{{{\mathbf{\upphi }}}};{{{\bf{X}}}})$ to calculate how the model describes the dataset using Jensen’s inequality,

$$\log {p}_{{{{\mathbf{\uptheta }}}}}\left({{{\bf{X}}}}\right)\ge {{{\mathcal{L}}}}\left({{{\mathbf{\uptheta }}}},{{{\mathbf{\upphi }}}};{{{\bf{X}}}}\right)=\mathop{\sum }\limits_{i=1}^{N}{{{\mathcal{L}}}}\left({{{\mathbf{\uptheta }}}},{{{\mathbf{\upphi }}}};{{{{\bf{x}}}}}^{(i)}\right),$$

where

1 $$\begin{array}{l}{{{\mathcal{L}}}}\left({{{\mathbf{\uptheta }}}},{{{\mathbf{\upphi }}}};{{{{\bf{x}}}}}^{(i)}\right)=-{D}_{\mathrm{KL}}\left({q}_{{{{\mathbf{\phi }}}}}\left({{{\bf{z}}}}| {{{{\bf{x}}}}}^{(i)}\right)\parallel {p}_{{{{\mathbf{\uptheta }}}}}({{{\bf{z}}}})\right)\\+{{\mathbb{E}}}_{{q}_{{{{\boldsymbol{\phi }}}}}\left({{{\bf{z}}}}| {{{{\bf{x}}}}}^{(i)}\right)}\left[\log {p}_{{{{\mathbf{\uptheta }}}}}\left({{{{\bf{x}}}}}^{(i)}| {{{\bf{z}}}}\right)\right],\end{array}$$

where *D*_KL_(*p*∣∣*q*) is the Kullback–Leibler divergence between distributions *p* and *q*. The first term on the right-hand-side is the regularization error, whereas the second term is the reconstruction error. Modeling this reconstruction error to suit the problem determines the structure of the latent space. Note that ELBO can be utilized as a measure to determine the optimal dimension of the latent space (that is, model selection)^44^. In this paper we refer to the negative value of ELBO as model loss or loss.

#### CNN-based encoder with skip connections

The RaptGen encoder network consists of a stack of convolutional layers with skip connections. Each character was first embedded into a 32-channel vector and went through seven convolutional layers with skip connections. Max pooling and fully connected layering then transform the vector into the distribution parameters of latent representation *q*_*ϕ*_(**z**∣**x**). The structure is shown in detail in Supplementary Section 5.

#### Profile HMM decoder model

For modeling insertions and deletions, we used the profile HMM as the decoder for RaptGen. The profile HMM is a model that outputs by probabilistically moving from state to state (Supplementary Fig. 2). The profile HMM consists of match (*M*), insertion (*I*) and deletion (*D*) states. Each state emits specific outputs introduced to represent multiple sequence alignments^45^. The match state has a high probability of emitting a particular character, the insertion state has an equal chance and the deletion state always emits a null character. These probabilities are called emission probabilities. The other probabilistic parameter is the transition probability. This defines the likeliness of transition from a state to the next state. In a profile HMM, the emission probability *e*_*S*_(*c*) is the probability of output character *c* from state *S*, and transition probability ${a}_{S,S^{\prime} }$ is the probability of changing state from *S* to $S^{\prime}$. These are defined as *e*_*S*_(*c*) = *p*(*c*∣*S*) and ${a}_{S,S^{\prime} }=p(S^{\prime} | S)$, respectively.

As profile HMM is a model in which the state transition depends only on the previous single state, the sequence probability *p*(**x**) can be written by using the Markov chain rule:

2 $$p\left({{{\bf{x}}}}\right)=\mathop{\sum}\limits_{\uppi }p\left({{{\bf{x}}}},\uppi \right)=p({x}_{0:L+1},{\uppi }_{{{\mbox{last}}}}={{M}}_{m+1}),$$

where π is the possible state path, π_last_ is the last state in the path, *L* is the length of the sequence, *x*_*j*:*k*_ is the subsequence of **x** from the *j*th character to the *k*th character on both ends, *x*_0_ is a null character that indicates the start of the sequence, *x*_*L*+1_ is a null character that indicates the end of the sequence, and *m* is the number of matching states in the model. It is computationally expensive to calculate the sequence probability for all possible paths. Introducing a forward algorithm can lower the computational cost to ${{{\mathcal{O}}}}(Lm)$. The forward algorithm consists of a forward variable defined as ${f}_{j}^{S}(i)=p({x}_{0:i},{\uppi }_{{{\mbox{last}}}}={S}_{j})$, and the probability can be calculated recurrently by

3 $$\begin{array}{lll}{f}_{k}^{M}(l)&=&{e}_{{M}_{k}}({x}_{l})\mathop{\sum}\limits_{S\in \{M,I,D\}}{a}_{{S}_{k-1},{M}_{k}}\;{f}_{k-1}^{S}(l-1),\\ {f}_{k}^{I}(l)&=&{e}_{I}({x}_{l})\mathop{\sum}\limits_{S\in \{M,I\}}{a}_{{S}_{k},{I}_{k}}\;{f}_{k}^{S}(l-1),\\ {f}_{k}^{D}(l)&=&\mathop{\sum}\limits_{S\in \{M,D\}}{a}_{{S}_{k-1},{D}_{k}}\;{f}_{k-1}^{S}(l).\end{array}$$

The emission probability of the insertion state does not depend on the position of the motif; therefore, it is set to a constant of one-quarter for RNA sequences. We set the probability to output the final end-of-sequence token *p*(*x*_*L*+1_∣*M*_*m*+1_) to 1.

#### Other tested decoders

Three probabilistic models were tested: the multicategorical model, the autoregressive model and profile HMM. The probabilistic models each have different sequence probability assignments. The multicategorical model assigns a categorical distribution to each position of the sequence. Given the representation vector **z** and the probability of the sequence **x**, *p*(**x**∣**z**) is calculated by $p({{{\bf{x}}}}| {{{\bf{z}}}})=\mathop{\prod }\nolimits_{i = 1}^{L}p({x}_{i}| z)=\mathop{\prod }\nolimits_{i = 1}^{L}{{{\rm{Cat}}}}({x}_{i}| {f}_{{{{\mathbf{\uptheta }}}}}({{{\bf{z}}}}))$, where Cat is a categorical distribution and *f*_**θ**_ is a neural network. The autoregressive model outputs a probability according to previous data. The probability of the sequence *p*(**x**∣**z**) is calculated by $p({{{\bf{x}}}}| {{{\bf{z}}}})=\mathop{\prod }\nolimits_{i = 1}^{L}p({x}_{i}| {x}_{0:i-1},{{{\bf{z}}}})=\mathop{\prod }\nolimits_{i = 1}^{L}{{{\rm{Cat}}}}({x}_{i}| {g}_{{{{\mathbf{\uptheta }}}}}({x}_{0:i-1},{{{\bf{z}}}}))$, where *g*_**θ**_ is a recurrent neural network. The architectures of networks *f*_**θ**_ and *g*_**θ**_ are described in Supplementary Section 5.

### Training techniques

State transition regularization was introduced to train RaptGen. Weighed regularization loss was also introduced for all VAEs, including RaptGen.

#### State transition regularization

A VAE can be trained with backpropagation by treating ELBO as a loss function. In addition to ELBO, a Dirichlet prior distribution was used on the transition probabilities to avoid unnecessary state transitions in the early rounds of training RaptGen. By penalizing transitions other than match-to-match at the beginning of the learning process, insertions and deletions are forced to occur less. This allows continuous motifs to be learned and lowers the probability of obtaining models with meaningless transitions traversing deletion states.

The probability of categorical variable **p** = {*p*_*k*_} sampled from a Dirichlet distribution is

4 $$\,{{\mbox{Dir}}}\,\left({{{\textbf{p}}}}| {{{\mathbf{\upalpha }}}}\right)=\frac{{{\Gamma }}\left(\mathop{\sum }\nolimits_{k = 1}^{K}{\alpha }_{k}\right)}{\mathop{\prod }\nolimits_{k = 1}^{K}{{\Gamma }}\left({\alpha }_{k}\right)}\mathop{\prod }\limits_{k=1}^{K}{p}_{k}^{{a}_{k}-1},$$

where **α** = {*α*_*k*_} is the Dirichlet distribution parameter. The regularization term is the sum of the log-odds ratio of the training probability from the matching state over each position *i*, defined as

5 $$\begin{array}{lll}{L}_{M}({{{{\textbf{p}}}}}_{i},e,r)&=&\log \left(\frac{\,{{\mbox{Dir}}}\,({{{{\boldsymbol{p}}}}}_{i}| {{{\boldsymbol{\alpha }}}}({w}_{m}))}{\,{{\mbox{Dir}}}\,({{{{\boldsymbol{p}}}}}_{i}| {{{\boldsymbol{\alpha }}}}(0))}\right)\\ &=&\log \left(\frac{{{\Gamma }}(3+{w}_{m})}{{{\Gamma }}(1+{w}_{m})}{({a}_{{M}_{i-1},{M}_{i}})}^{{w}_{m}}\times \frac{1}{{{\Gamma }}(3)}\right)\\ &=&\log \left(\frac{(2+{w}_{m})(1+{w}_{m})}{2}{({a}_{{M}_{i-1},{M}_{i}})}^{{w}_{m}}\right),\end{array}$$

where **p**_*i*_ is $[{a}_{{M}_{i-1},{M}_{i}}\,{a}_{{M}_{i-1},{I}_{i}}\,{a}_{{M}_{i-1},{D}_{i}}]$ which indicates the transition probabilities from the *i*th matching state, and **α**(*w*_*m*_) = [1 + *w*_*m*_ 1 1] is the parameter representing the induction weight *w*_*m*_. To make this loss zero at a specific round *R*, *w*_*m*_ was set to 4(1 − *e*/*R*), where *e* is the training epoch. This regularization term was added to the ELBO during training.

#### Weighted regularization loss

The scaling param eter for the regularization was introduced to train the VAE. Scaling the regularization term of the loss function of the VAE to minimize the value in the early epoch of training improves latent embedding^46^. The scale is defined as *e*/*E*, where *e* is the training epoch, and *E* is the maximum number of epochs to have scaling. After the *E* epochs of training have finished, the scale is set to 1.

#### Training settings

All sequences in the training set were filtered first. Sequences with exact matching adapters, exact matching sequence design lengths, and sequences read more than once remained. The sequences were split into training and test datasets in a 9:1 ratio. The model with the smallest test loss was selected through iterations. For the weighted regularization loss, the maximum number to have scaling *E* was set to 50. The state transition regularization parameter *R* was set to 50 for the profile HMM decoder. Adam was used as the training optimizer with default parameters^47^. All of the networks were trained up to 2,000 epochs with early stopping when the test loss was not updated for 50 epochs.

### RaptGen evaluation

#### Simulation data

For the simulation data shown in Fig. 2a, ten different motif sequences of length ten were generated and single nucleotide modification with a 10% error rate was added. In other words, each motif sequence had a 3.33 … % chance of deletion, insertion or modification at a specific position. After this procedure, sequences were randomly extended to reach 20 nt by adding nucleotides to the right and the left. We made 10,000 sequences in total, with no duplication.

For the simulation data shown in Fig. 3a, sequences containing paired motifs were generated. Two 5 nt motifs were made, and then one of the motifs was randomly deleted at a probability of 25% each. If both motifs remained, 2 to 6 nt were randomly inserted between the left and right motifs. Sequences were then randomly extended to reach 20 nt, and 5,000 of these sequences were generated.

#### SELEX data

SELEX data used in this study were obtained previously^20^. The sequences are available as DRA009383 and DRA009384, which we call datasets A and B, respectively. These SELEX were conducted using a conventional selection method. Briefly, the target proteins were immobilized on beads. After washing, bound RNA was recovered and amplified using reverse-transcription-PCR. Dataset A, targeting human transglutaminase 2, consists of nine SELEX rounds from 0 to 8, and Dataset B, targeting human integrin alpha V beta 3, consists of four rounds from 3 to 6. The round with the smallest unique ratio *U*(*T*) with the restriction of *U*(*T*) > 0.5 was used, defined as

6 $$U(T)=\frac{| \{{{{\bf{x}}}}| {{{\bf{x}}}}\in {{{\mathcal{D}}}}(T)\}| }{| {{{\mathcal{D}}}}(T)| },$$

where ${{{\mathcal{D}}}}(T)$ are the whole sequences, read in round *T*. The fourth round was selected for each dataset.

### RaptGen applications in aptamer discovery

#### GMM for initial sequence selection

We used the GMM for initial sequence selection from the obtained latent space. To efficiently select ten points to be evaluated, GMM was run 100 times with ten components, and the mean vectors of the model with the best evidence (likelihood) were selected.

#### Surface plasmon resonance assay

The surface plasmon resonance assays were performed using a Biacore T200 instrument (GE Healthcare) as described previously with slight modifications^20^. The target proteins of datasets A and B were human recombinant transglutaminase 2 (R&D systems, catalogue no. 4376-TG) and human recombinant integrin alpha V beta 3 (R&D systems, catalogue no. 3050-AV), respectively. Aptamers were prepared with fixed primer regions and 16-mer poly(A)-tails as follows: 5′–GGGAGCAGGAGAGAGGUCAGAUG–(variable sequence)–CCUAUGCGUGCUAGUGUGA–(polyA)–3′ for dataset A and 5′–GGGAGAACUUCGACCAGAAG–(variable sequence)–UAUGUGCGCAUACAUGGAUCCUC–(polyA)–3′ for Dataset B. Previously reported aptamers were used as positive controls. All evaluated sequences are listed in Supplementary Section 2 (Supplementary Table 3). Aptamers were prepared by in vitro transcription using a mutant T7 RNA polymerase and 2′-fluoro-pyrimidine NTPs. The running buffer consisted of 145 mM NaCl, 5.4 mM KCl, 0.8 mM MgCl_2_, 1.8 mM CaCl_2_, 0.05% Tween20 and 20 mM Tris-HCl (pH 7.6). A 5′-biotinylated dT16 oligomer was immobilized to both active and reference flow cells of the streptavidin sensor chip (BR100531, GE Healthcare). The poly(A)-tailed RNA was captured in the active flow cell by complementary hybridization at a concentration of 300 nM and a flow rate of 20 μl min^−1^, with an association time of 60 s. The proteins were injected into the flow cells of the sensor chip at a concentration of 50 nM and a flow rate of 20 μl min^−1^, with an association time of 60 s. To regenerate the sensor chip, bound aptamers were completely removed by injecting 6 M urea. Data were obtained by subtracting the reference flow cell data from the active flow cell data. The ratio of the protein-binding level to aptamer-capturing level was used as binding activity. Percent relative binding activities of positive control aptamers are shown in the results and discussion section. For normalization of Dataset A, the cycle number-dependent reduction of control aptamer binding was estimated.

#### Multipoint Bayesian optimization via local penalization

Bayesian optimization uses both the search for sequences that have not been explored to a reasonable extent and the utility of utilizing sequences with known affinity to select the next sequence for evaluation. The local penalization function is a method that can determine the multipoint expected improvement of candidates by considering the smoothness of the potential function^48^. As it converges faster than qEI^49^ and other methods for simultaneous optimization. We used this method to perform multipoint optimization. Implementation was performed with the GPyOpt package^50^.

### Reporting summary

Further information on research design is available in the Nature Research Reporting Summary linked to this article.

### Supplementary information

Supplementary Information Supplementary Figs. 1–6, Tables 1 and 2, and Sections 1–9

Reporting Summary

Supplementary Data The detailed results for secondary structure analyses.

### Source data

Source Data Fig. 2 Statistical Source Data

Source Data Fig. 3 Statistical Source Data

Source Data Fig. 4 Statistical Source Data

Source Data Fig. 5 Statistical Source Data

Source Data Fig. 6 Statistical Source Data

## Data Availability

The HT-SELEX sequences are available as DRA009383 (Dataset A) and DRA009384 (Dataset B) in DDBJ. Source Data are provided with this paper.

## Code availability

The RaptGen program is available via ref. ^51^.

## References

[1] Ni, S. et al. Recent progress in aptamer discoveries and modifications for therapeutic applications. *ACS Appl. Mater. Interfaces* **13**, 9500–9519 (2020). 10.1021/acsami.0c05750 32603135

[2] Adachi T, NakamuraAptamers Y (2019). A review of their chemical properties and modifications for therapeutic application. Molecules.

[3] Song S, Wang L, Li J, Fan C, Zhao J (2008). Aptamer-based biosensors. Trends Anal. Chem..

[4] Zhou W, Huang P-JJ, Ding J, Liu J (2014). Aptamer-based biosensors for biomedical diagnostics. Analyst.

[5] Eyetech Study Group. (2002). Preclinical and phase 1A clinical evaluation of an anti-VEGF pegylated aptamer (EYE001) for the treatment of exudative age-related macular degeneration. Retina.

[6] Ciesiolka J, Gorski J, Yarus M (1995). Selection of an RNA domain that binds Zn^2+^. RNA.

[7] Tombelli S, Minunni M, Luzi E, Mascini M (2005). Aptamer-based biosensors for the detection of HIV-1 TAT protein. Bioelectrochemistry.

[8] Jolma A (2013). DNA-binding specificities of human transcription factors. Cell.

[9] Binning JM (2013). Development of RNA aptamers targeting Ebola virus VP35. Biochemistry.

[10] Baker BR (2006). An electronic, aptamer-based small-molecule sensor for the rapid, label-free detection of cocaine in adulterated samples and biological fluids. J. Am. Chem. Soc..

[11] Labib M (2012). Aptamer-based viability impedimetric sensor for bacteria. Anal. Chem..

[12] Tuerk C, Gold L (1990). Systematic evolution of ligands by exponential enrichment: RNA ligands to bacteriophage T4 DNA polymerase. Science.

[13] Ellington AD, Szostak JW (1990). In vitro selection of RNA molecules that bind specific ligands. Nature.

[14] Zhao Y, Granas D, Stormo GD (2009). Inferring binding energies from selected binding sites. PLoS Comput. Biol..

[15] Jolma A (2010). Multiplexed massively parallel SELEX for characterization of human transcription factor binding specificities. Genome Res..

[16] Kupakuwana GV, Crill JE, McPike MP, Borer PN (2011). Acyclic identification of aptamers for human alpha-thrombin using over-represented libraries and deep sequencing. PLoS ONE.

[17] Jiang P (2014). MPBind: a meta-motif-based statistical framework and pipeline to predict binding potential of SELEX-derived aptamers. Bioinformatics.

[18] Caroli J, Taccioli C, Fuente ADL, Serafini P, Bicciato S (2016). APTANI: a computational tool to select aptamers through sequence-structure motif analysis of HT-SELEX data. Bioinformatics.

[19] Caroli J, Forcato M, Bicciato S (2020). APTANI2: update of aptamer selection through sequence-structure analysis. Bioinformatics.

[20] Ishida R (2020). RaptRanker: in silico RNA aptamer selection from HT-SELEX experiment based on local sequence and structure information. Nucl. Acids Res..

[21] Kim N, Izzo JA, Elmetwaly S, Gan HH, Schlick T (2010). Computational generation and screening of RNA motifs in large nucleotide sequence pools. Nucl. Acids Res..

[22] Hoinka J (2015). Large scale analysis of the mutational landscape in HT-SELEX improves aptamer discovery. Nucl. Acids Res..

[23] Zhou Q, Xia X, Luo Z, Liang H, Shakhnovich E (2015). Searching the sequence space for potent aptamers using SELEX in silico. J. Chem. Theory Comput..

[24] Hiller M, Pudimat R, Busch A, Backofen R (2006). Using RNA secondary structures to guide sequence motif finding towards single-stranded regions. Nucl. Acids Res..

[25] Dao P (2016). AptaTRACE elucidates RNA sequence-structure motifs from selection trends in HT-SELEX experiments. Cell Syst..

[26] Hoinka J, Zotenko E, Friedman A, Sauna ZE, Przytycka TM (2012). Identification of sequence-structure rna binding motifs for SELEX-derived aptamers. Bioinformatics.

[27] Alipanahi B, Delong A, Weirauch MT, Frey BJ (2015). Predicting the sequence specificities of DNA-and RNA-binding proteins by deep learning. Nat. Biotechnol..

[28] Hassanzadeh, H. R. & Wang, M. D. Deeperbind: enhancing prediction of sequence specificities of DNA binding proteins. In *2016 IEEE International Conference on Bioinformatics and Biomedicine (BIBM)* 178–183 (IEEE, 2016). 10.1109/bibm.2016.7822515 PMC7302108 32551184

[29] Pan X, Rijnbeek P, Yan J, Shen H-B (2018). Prediction of RNA-protein sequence and structure binding preferences using deep convolutional and recurrent neural networks. BMC genomics.

[30] Hinton GE (2009). Deep belief networks. Scholarpedia.

[31] Kingma, D. P. & Welling, M. Auto-encoding variational bayes. Preprint at https://arxiv.org/abs/1312.6114 (2013).

[32] Goodfellow I (2014). Generative adversarial nets. Adv. Neural Information Process. Syst..

[33] Im J, Park B, Han K (2019). A generative model for constructing nucleic acid sequences binding to a protein. BMC Genomics.

[34] Killoran, N., Lee, L. J., Delong, A., Duvenaud, D. & Frey, B. J. Generating and designing DNA with deep generative models. Preprint at https://arxiv.org/abs/1712.06148 (2017).

[35] Kusner, M. J., Paige, B. & Hernández-Lobato, J. M. Grammar variational autoencoder. Preprint at https://arxiv.org/abs/1703.01925 (2017).

[36] Gómez-Bombarelli R (2018). Automatic chemical design using a data-driven continuous representation of molecules. ACS Central Sci..

[37] Hochreiter S, Schmidhuber J (1997). Long short-term memory. Neural Comput..

[38] Lozupone C, Changayil S, Majerfeld I, Yarus M (2003). Selection of the simplest RNA that binds isoleucine. RNA.

[39] Gonzalez, J., Longworth, J., James, D. C. & Lawrence, N. D. Bayesian optimization for synthetic gene design. Preprint at https://arxiv.org/abs/1505.01627 (2015).

[40] Hamada M (2016). Rtools: a web server for various secondary structural analyses on single RNA sequences. Nucl. Acids Res..

[41] Lorenz R (2011). ViennaRNA Package 2.0. Algorithms Mol Biol.

[42] Sakakibara Y (1994). Stochastic context-free grammers for tRNA modeling. Nucl. Acids Res..

[43] Bell DR (2020). In silico design and validation of high-affinity RNA aptamers targeting epithelial cellular adhesion molecule dimers. Proc. Natl Acad. Sci. USA.

[44] Corduneanu, A. & Bishop, C. Variational bayesian model selection for mixture distributions. In *Proc. 8th International Conference on Artificial Intelligence and Statistics* 27–34 (Morgan Kaufmann, 2001).

[45] Krogh A, Brown M, Mian IS, Sjolander K, Haussler D (1994). Hidden Markov models in computational biology. applications to protein modeling. J. Mol. Biol..

[46] Bowman, S. R. et al. Generating sentences from a continuous space. Preprint at https://arxiv.org/abs/1511.06349 (2015).

[47] Kingma, D. P. & Ba, J. Adam: a method for stochastic optimization. Preprint at https://arxiv.org/abs/1412.6980 (2014).

[48] González, J., Dai, Z., Hennig, P. & Lawrence, N. Batch Bayesian optimization via local penalization. In *Proc. 19th International Conference on Artificial Intelligence and Statistics* 648–657 (PMLR, 2016).

[49] Ginsbourger, D, Le Riche, R. & Carraro, L. Kriging is well-suited to parallelize optimization. In *Computational Intelligence in Expensive Optimization Problems* 131–162 (Springer, 2010).

[50] The GPyOpt authors. *GPyOpt: A Bayesian Optimization Framework in Python* (GitHub, 2016); http://github.com/SheffieldML/GPyOpt

[51] The RaptGen authors. *Raptgen Version 1.0* (Zenodo, 2022); 10.5281/zenodo.6470866

[52] Auer P (2002). Using confidence bounds for exploitation-exploration trade-offs. J. Mach. Learn. Res..

