# Supplementary material for: Generative aptamer discovery using RaptGen
Source: Nat Comput Sci. 2022 Jun 2;2(6):378–86. doi: 10.1038/s43588-022-00249-6 (PMC10766510; doi:10.1038/s43588-022-00249-6)
Supplement: Supplementary file 1 — Supplementary Figs. 1–6, Tables 1 and 2, and Sections 1–9 [file 43588_2022_249_MOESM1_ESM.pdf]

nature portfolio

https://doi.org/10.1038/s43588-022-00249-6

Supplementary information

Generative aptamer discovery using RaptGen

In the format provided by the authors and unedited

Supplementary Information of “Generative aptamer discovery using RaptGen”

Natsuki Iwano [1], Tatsuo Adachi [2], Kazuteru Aoki [2], Yoshikazu Nakamura [2] and Michiaki Hamada [1,3,4*]

1 Graduate School of Advanced Science and Engineering, Waseda University, Tokyo, Japan
2 RIBOMIC, inc., Tokyo, Japan
3 Computational Bio Big-Data Open Innovation Laboratory (CBBD-OIL), National Institute of Advanced Industrial Science and Technology (AIST), Tokyo, Japan
4 Graduate School of Medicine, Nippon Medical School, Tokyo, Japan

Abbreviation

The following is the list of abbreviation utilized in the main paper.

- AR: Auto-regressive
- BO: Bayesian optimization
- DBN: Deep belief network
- ELBO: Evidence lower bound
- GAN: Generative adversarial network
- GMM: Gaussian mixture model
- GP-UCB: Gaussian process upper confidence bound
- HMM: Hidden Markov model
- HT-SELEX: High-throughput SELEX
- LSTM: Long short-term memory
- MC: Multi-categorical
- PFM: Position frequency matrix
- SCFG: Stochastic context-free grammar
- SELEX: Systematic Evolution of Ligands by EXponential enrichment
- SPR: Surface plasmon resonance
- VAE: Variational auto-encoder

Supplementary Figures

*Corresponding author: Tel: +81 3 5286 3130; Fax: +81 3 5286 3130; Email: mhamada@waseda.jp

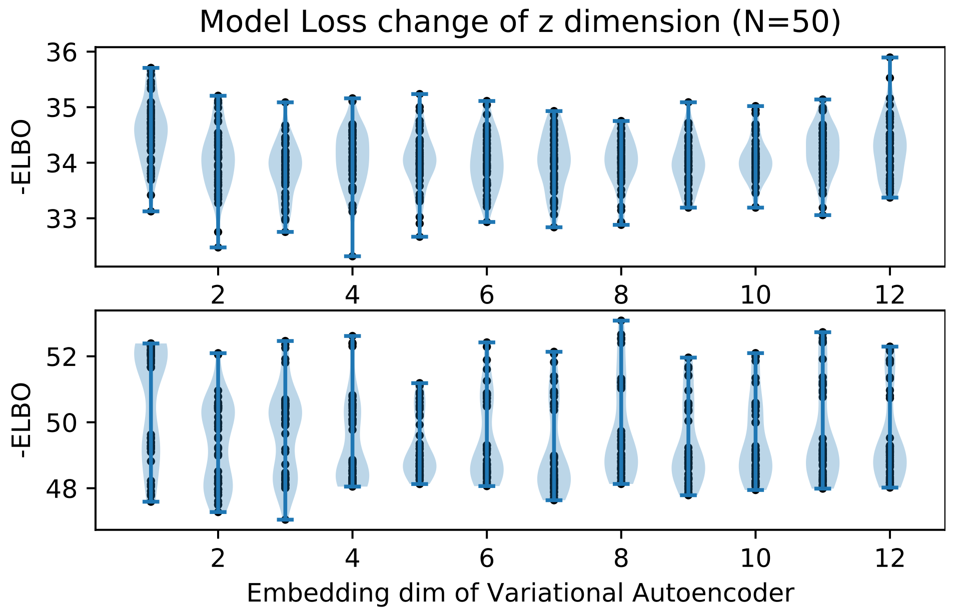

Supplementary Figure 1. Model loss (i.e. minus value of ELBO in Eq. (1) in the main paper) over different latent dimensions. Smaller values indicate better models. The model was trained using Dataset A (top) and B (bottom) with different dimension numbers. The minimum loss of training was plotted. Data were obtained from 50 runs with random parameter initialization.

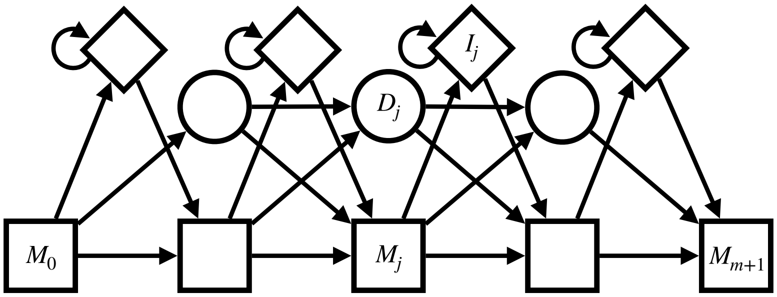

Supplementary Figure 2. Profile HMM. The squares, circles, and diamonds represent the match M, deletion D, and insertion I states, respectively. The arrows represent possible transition directions of the states. The insertion and deletion states cannot go back and forth between each other. Each matching state emits a character, while the start matching state M_0 and the end matching state M_{m+1} emits a null character.

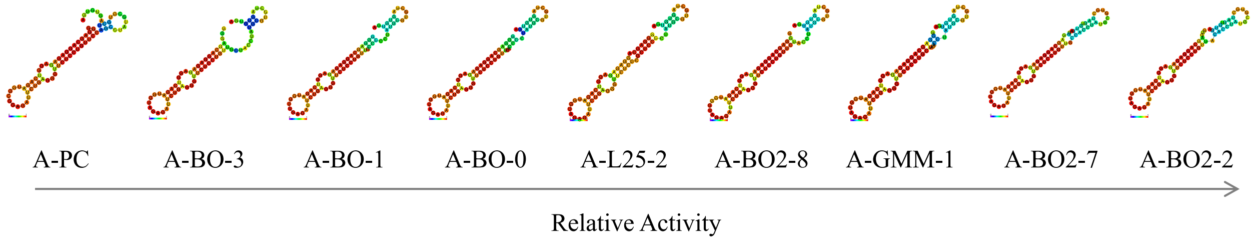

Supplementary Figure 3. RNA secondary structures (predicted by CentroidFold [1]) of newly obtained aptamers for Dataset A. The leftmost structure (A-PC) is the positive control aptamer, and the structures on the right show higher relative activity (compared with the positive control). In each structure, warmer colors indicate higher base-pairing probabilities (for base-pairs) and loop probabilities (for un-pair nucleotides); these probabilities are computed based on a Boltzmann distribution of RNA secondary structures. See Supplementary Figure 4 for Dataset B.

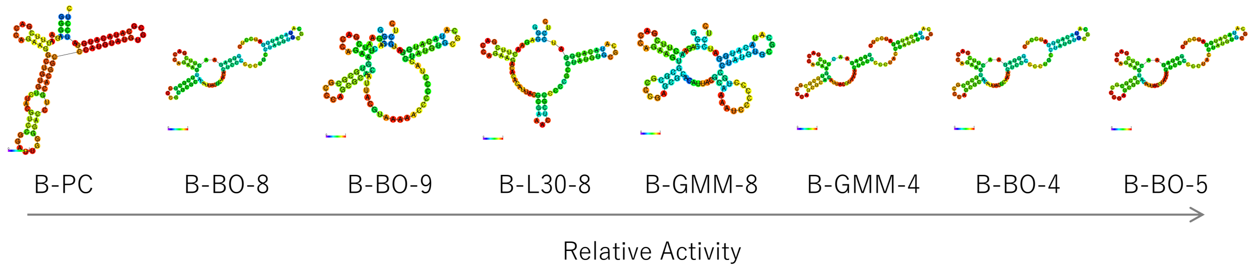

Supplementary Figure 4. RNA secondary structures (predicted using CentroidFold [1]) of newly-obtained aptamers for Dataset B. The leftmost structure (B-PC) is the positive control aptamer, and structures on the right show higher relative activity (compared with the positive control). In each structure, warmer colors indicate higher base-pairing probabilities (for base-pairs) and loop probabilities (for un-pair nucleotides). The probabilities were computed based on a Boltzmann distribution of RNA secondary structures.

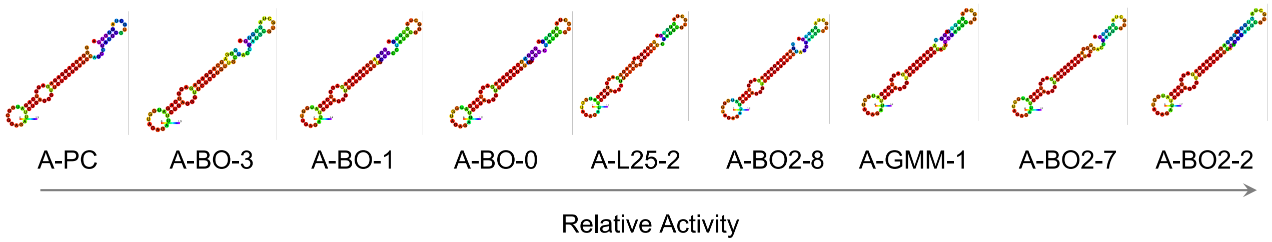

Supplementary Figure 5. RNA secondary structures (predicted using RNAfold [2]) of newly-obtained aptamers for Dataset A. The leftmost structure (A-PC) is the positive control aptamer, and structures on the right show higher relative activity (compared with the positive control). In each structure, warmer colors indicate higher base-pairing probabilities (for base-pairs) and loop probabilities (for un-pair nucleotides). The probabilities were computed based on a Boltzmann distribution of RNA secondary structures.

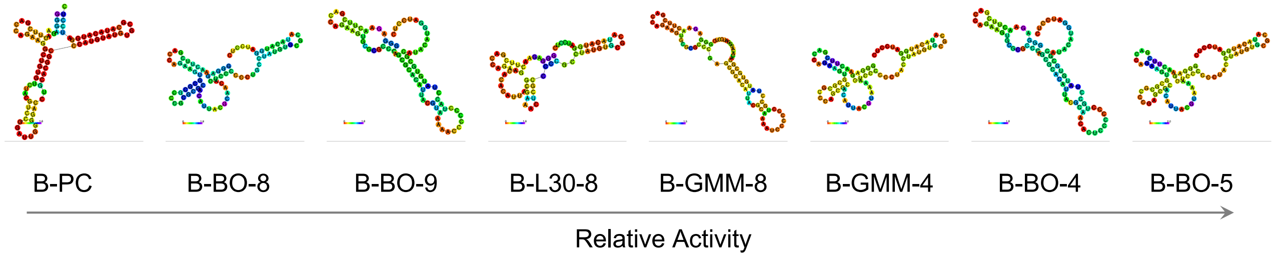

Supplementary Figure 6. RNA secondary structures (predicted using RNAfold [2]) of newly-obtained aptamers for Dataset B. The leftmost structure (B-PC) is the positive control aptamer, and structures on the right show higher relative activity (compared with the positive control). In each structure, warmer colors indicate higher base-pairing probabilities (for base-pairs) and loop probabilities (for un-pair nucleotides). The probabilities were computed based on a Boltzmann distribution of RNA secondary structures.

Supplementary Tables

Supplementary Table 1. The sequences derived by RaptGen from centers of GMM. The log probabilities of the sequences generation from Profile HMM are shown. Aptamer activities were evaluated by SPR assay. Relative activities against respective positive control are shown and the activity higher than the control are shown in bold font. The edit distance to the nearest sequence within the whole sequencing data were calculated.

ID	Log probability	Relative activity	Edit distance
A-GMM-0	-15.8	79.0	4
A-GMM-1	-13.3	**107.1**	4
A-GMM-2	-34.9	-3.6	9
A-GMM-3	-16.7	7.0	3
A-GMM-4	-19.1	15.9	6
A-GMM-5	-30.1	-0.8	7
A-GMM-6	-14.9	42.2	4
A-GMM-7	-17.0	30.9	5
A-GMM-8	-18.7	33.8	5
A-GMM-9	-14.5	74.3	3
B-GMM-0	-47.0	-13.2	15
B-GMM-1	-35.9	21.9	11
B-GMM-2	-29.9	-9.3	10
B-GMM-3	-23.9	74.2	8
B-GMM-4	-23.3	**229.1**	7
B-GMM-5	-26.8	-30.5	9
B-GMM-6	-27.5	-28.5	8
B-GMM-7	-26.4	-7.3	9
B-GMM-8	-24.5	**190.7**	8
B-GMM-9	-23.0	-7.3	8

Supplementary Table 2. The top three sequences selected by BO and their nearest tested sequence in the embedded space. Profile HMM obtained by BO were reconstructed into a sequence by deriving the maximum probable sequence. Binding activities of the reconstructed aptamers (sequences) were evaluated by SPR experiment. Top 3 binding aptamers and their activities are shown in alignment view with nearest GMM clones. The Hyphen indicates gap and the lowercase letter indicates substitutions. Relative activities of A-GMM-1 and B-GMM-4 were redisplayed from Figure 4 in the main paper for comparison. BO2 denotes a second round of BO.

ID	maximum probable sequence	Relative Activity
A-GMM-1	-GTAGAGATTCTGAGGGTTCTCCTGCT-ATA	107.1
A-BO-0	-GTAGAGATTCTGAGGGTTCTCCTGtTGAcc	102.5
A-BO-1	-GTtGAGATTCTGAGGGTTCTCCTGtTGccc	101.2
A-BO-3	-GTAGAGATTCTGAGGGTTCTCCTGtTGcTA	100.6
A-BO2-2	-GTAGAGATTCTGAGGGTTCTCCTGtTAc-A	119.8
A-BO2-7	-GTAGAGATTCTGAGGGTTCTCCTGtTGccA	116.2
A-BO2-8	-GTAGAGATTCTGAGGGTTCTCCTGtTGcTA	105.8
B-GMM-4	TGCGCGCCC-AGCGCACATTACGTAAAACTCCCCCCTACC	229.1
B-BO-5	TGCGCGCCCGAGCGCACATTACGTAAAACTCCCCCCTACC	245.2
B-BO-4	TGCGCGCCC-AGCGCACATTACGcAAcACTCCCCCTgCC	231.0
B-BO-9	TGCGCGCCC-AGCGCACATTACGTAAAA-aCCCCCCTACC	151.6

Supplementary Sections

Supplementary Section 1 Method to Create Sequence Logo

To create a sequence logo for the given profile HMM, we calculated the most probable path of the profile HMM. The most probable path was iteratively acquired using the following pseudocode.

Algorithm 1 Sequence logo generation

$M[0] \leftarrow 1, D[0] \leftarrow 0$
$I[0] \leftarrow a_{M_0,I_0}$
foreach $i \in [1, m]$ **do**
  $M[i] \leftarrow \max(a_{M_{i-1},M_i} M[i-1], a_{I_{i-1},M_i} I[i-1], a_{D_{i-1},M_i} D[i-1])$
  $I[i] \leftarrow a_{M_i,I_i} M[i]$
  $D[i] \leftarrow \max(a_{M_{i-1},D_i} M[i-1], a_{D_{i-1},D_i} D[i-1])$
end foreach
$M[m+1] \leftarrow \max(a_{M_m,M_{m+1}} M[m], a_{I_m,M_{m+1}} I[m], a_{D_m,M_{m+1}} D[m])$
traceback and acquire most probable path

When calculating the probability of insertion state I, the state's recurrency was ignored because the probability of staying on I recurrently is lower than that of immediately moving to the next state. After the most probable path was achieved, the sequence logo was written according to the emission probability of each state using WebLogo technology [3, 4]. The overall height R_i at state S is defined as

$$R_S = \log_2(|C|) - (H_S + e_n)$$

where $|C|$ is the number of characters (typically 4 for RNA), H_S is the uncertainty at state S, and e_n is the correction factor. The correction factor e_n adjusts the result when there are a few sample sequences. In our setting, we set e_n to 0. The uncertainty H_S is defined as

$$H_S = - \sum_{b \in \{\mathrm{A,U,G,C}\}} e_S(b) \log_2 e_S(b)$$

where b is one of the bases (A, U, G, C) and $e_S(b)$ is the probability of emitting base b from state S. The height of the base at a certain state $h_S(b)$ is defined as

$$h_S(b) = e_S(b) R_S.$$

The sequence logo was written using $h_S(b)$, placing the higher probable base at the bottom and the state path sequence from left to right.

Supplementary Section 2 Sequence Obtained in the Present Study

The sequences obtained in the present study are shown in Supplementary Table 3. The ID is named after a rule of dataID, length of the trained model, the method to select the sequence, and the index of the sequence. max_seq indicates that the sequence was the most probable sequence emitted from the most probable state path of the given profile HMM. Relative activity shows % relative binding activities of positive controls assessed by the SPR experiment. The positive control sequences are as follows: A-PC is equal to Data1-11, and B-PC is equal to Data2-1 in our previous report [5] .

Supplementary Table 3. The sequences obtained through data are shown. Log probability is the probability that the sequence was obtained from a certain point of space. For GMM-, it was obtained in the initial sequence selection, and for BO-, it was obtained in the next candidate selection procedure.

ID	maximum probable sequences	length	log probability	relative activity
A-PC	GCGGAGAUUCUGAGGGUUCUCCUGUUCCAG	30	-	(100)
A-L20-GMM-0	CUCGAGAUUCUGAGGGUUCUGCAUA	25	-13.8	-0.7
A-L20-GMM-1	AAGAAAUUAGAUACUAGUAAAUACGACAUA	30	-36.9	-4.9
A-L20-GMM-2	UGUGCGGAUAUGCUGAGUUUCUGC	24	-17.9	-4.9
A-L20-GMM-3	AACGAGAGAUACAACCUGUGCGUGCC	26	-17.0	8.8
A-L20-GMM-4	AUCGAGAGAUGGUAGACCCUGUGCG	25	-14.6	-2.7
A-L20-GMM-5	CAAAGACGUGCAAGCCUCGUCUACGGU	27	-21.8	-1.5
A-L20-GMM-6	UCUGAGGGUCCUGACAACAGAAACA	25	-16.2	-0.7
A-L20-GMM-7	UACGAGAGAUGUAGCCUGUAUGUGCU	26	-16.3	11.9
A-L20-GMM-8	CAGUAAGGUUUCGACACUCUUCUACUUA	28	-28.9	-2.4
A-L20-GMM-9	AACGAGAGAUGUUGCCUGCGUGUUGC	26	-20.8	4.0
A-L25-GMM-0	CAUCAAUAAUAAGAAUAACAAAAUUUCAAA	30	-35.5	-1.1
A-L25-GMM-1	CAACUACGAGAGAUGUAGCCUGGA	24	-15.4	-6.1
A-L25-GMM-2	GUAGAGAUUCUGAGGGUUCUCCCGCUCC	28	-13.7	103.6
A-L25-GMM-3	GGCGGUGAUGUAGAAACGGUUGAGGUUAA	29	-25.0	-2.1
A-L25-GMM-4	AUACGAGAGAUGUAGCCUGUGUCGUAGAA	29	-14.7	-4.0
A-L25-GMM-5	AACGAGAGAUGGUAGACCGUUGUGGAU	27	-15.3	63.2
A-L25-GMM-6	CACGCGGGUUUCACACUCUAUGAAUGA	27	-19.5	-0.1
A-L25-GMM-7	CGUAGGGAAUCUGAGGGGUCUCCCGCCCU	29	-19.7	21.5
A-L25-GMM-8	AACGGUAACACGUGCAAGCCUGUUAUUAU	29	-19.3	-4.3
A-L25-GMM-9	AUUACGAGAGAUACAGCCUAUAUCGUAGCA	30	-15.4	15.4
A-GMM-0	AACGAGAGAUGGUAGACCUAUCUUUUAGCC	30	-15.8	79.0
A-GMM-1	GUAGAGAUUCUGAGGGUUCUCCUGCUAUA	29	-13.3	107.1
A-GMM-2	UUUUAUAAAAAAGUGUUUAAAAAAGAUUCA	30	-34.9	-3.6
A-GMM-3	GUAGAAAUUACGAGAGAUGUCGCCUUUGA	29	-16.7	7.0
A-GMM-4	GGGGGUGCAGUAGAAUUGUCGAGUUUCUG	29	-19.1	15.9
A-GMM-5	AAUACCCGGGGUUUUCACACAUAUAAUUCA	30	-30.1	-0.8
A-GMM-6	AUACGAGAGAUGUAGCCUUUUUUCUGACUU	30	-14.9	42.2
A-GMM-7	AGUACGAGAGAUACAGCCUUUUUCCUGCUU	30	-17.0	30.9
A-GMM-8	GGUAGCAGAUGCUGAGGGGUCUCCUGAUGC	30	-18.7	33.8
A-GMM-9	GUCGAGAUUCUGAGGGUUCUCCUGUUAACC	30	-14.5	74.3
A-BO-0	GUAGAGAUUCUGAGGGUUCUCCUGUUGACC	30	-14.4	102.5
A-BO-1	GUUGAGAUUCUGAGGGUUCUCCUGUUGCCC	30	-14.4	101.2
A-BO-2	AACAAGAGAUGGUAGACCUAUCUCUUACCC	30	-15.6	69.4
A-BO-3	GUAGAGAUUCUGAGGGUUCUCCUGUUGCUA	30	-14.3	100.6
A-BO-4	AACGAGAGAUGGUAGACCUAUCUUUUAGCC	30	-16.2	76.0
A-BO-5	GUCGAGAUUCUGAGGGUUCUCCUGGUGACC	30	-14.4	74.6
A-BO-6	AACGAGAGAUGGUAGACCUAUUUUUUAGUC	30	-16.2	73.1
A-BO-7	AAUGAGAUUCUGAGGGGUCUCCUGUUGCCA	30	-14.4	95.1
A-BO-8	AUACGAGAGAUGUAGCCUUUUUUCUUACUU	30	-15.2	40.8

Supplementary Table 3 continued from the previous page

ID	maximum probable sequences	length	log probability	relative activity
A-BO-9	AUACGAGAGAUGUAGCCUUUUUACCGACCU	30	-14.7	15.0
A-BO2-0	AACGAGAGAUGGUGGGCCUUUCUUUUAGCC	30	-15.2	62.5
A-BO2-1	UAUGAAAUGCUGAGGGGUCUCCUGUUGCCA	30	-13.8	97.4
A-BO2-2	GUAGAGAUUCUGAGGGUUCUCCUGUUACA	29	-13.9	119.8
A-BO2-3	AAUACGAGAGAUGUAGCCUUUUCACCGCCCU	31	-16.9	20.3
A-BO2-4	AACGAGAGUCCGUAGGCCUUUUUAUUAGCA	30	-18.3	-4.9
A-BO2-5	GUCGAGAUUCUGAGAGCUUCUCCUGCUACA	30	-13.6	64.2
A-BO2-6	AACGAGAGCGUGUAGGCCUAUUUUUUAGUC	30	-22.9	-3.9
A-BO2-7	GUAGAGAUUCUGAGGGUUCUCCUGUUGCCA	30	-14.4	116.2
A-BO2-8	GGUAGAGAUUCUGAGGGUUCUCCUGUUCUA	30	-16.1	105.8
A-BO2-9	AACCGUGGGUCUUUACAAAAAUUUAUUUCG	30	-25.2	-0.8
B-PC	GCUGUGUCUACGUCCGGAUUGGGGACCUGCACGGCCCAUG	40	-	(100)
B-L30-GMM-0	ACUCACAUUACGUAAAAAUCGCCCCUACC	29	-14.9	6.4
B-L30-GMM-1	UGGAUACGCAAAAGCUCCCCCUGCCUUACA	30	-21.6	-16.1
B-L30-GMM-2	UAAAACAGCUGCCCCCCCCCCAUCUGACCCGACGACCAAC	40	-50.7	-14.6
B-L30-GMM-3	UACGACCAGCUACGCAACAGUUCUCCCUGCCUGA	34	-20.7	-22.9
B-L30-GMM-4	UUCGCUACGCGAAAGUUCCCCCCGCCUGGCG	31	-21.0	-12.4
B-L30-GMM-5	UUCGCUACGCUAAAGCUCUCCCAGCCUGGCG	31	-21.7	-21.6
B-L30-GMM-6	ACUCACAUUACGCAAAACUCGCCCCUGCC	29	-14.7	86.9
B-L30-GMM-7	UCUACGUCAAACUCGCCCCGACCUGGCG	28	-20.0	-21.9
B-L30-GMM-8	ACACACAUUACGCGAAACUCGCCCCCGCC	29	-14.3	183.3
B-L30-GMM-9	UUCGCUACGCAAAAGUUCCCCCUGCCUGGCG	31	-20.5	-19.7
B-L35-GMM-0	UGCUCGACACUACGCAACACUUCCCCCUGCCUGACAUA	38	-31.0	-12.1
B-L35-GMM-1	UCCGCACGCCAGCGCACAUUACGUAAAGAUCGCCCUACC	39	-23.9	-5.2
B-L35-GMM-2	UGCACGACGCUACGCCAAACUCCCCCCGGCCUGAUAAA	38	-31.5	57.7
B-L35-GMM-3	UAACACAGCCCCACCCCUCGGACGACCGGAGGAAUAAAAA	40	-44.8	-14.9
B-L35-GMM-4	UGCUCUAGCUACGCGAAAAUCCCCCCCGCCUGCAUGCGCACA	42	-35.2	23.0
B-L35-GMM-5	UCCGCCCGCCAGCGCACAUUACGCAAAGAUCGCCCUGCC	39	-24.8	-25.7
B-L35-GMM-6	UUCGCUAGCGUACGCAAAACUCCCCCCUGCCUUGCAUGCGCUUA	44	-40.3	-16.9
B-L35-GMM-7	UACCACGCCCGCGCACAUUACGCGAAGAUCGCCCCGCC	38	-21.9	-22.4
B-L35-GMM-8	UCCGCCAACCCCCCCCCUCCCCCACCCCCCCACCUCCAAA	40	-47.5	-22.5
B-L35-GMM-9	UGCGCGUCACUACGCUAAAAUCCCCCCAGCCUGACAAA	38	-31.9	-10.0
B-GMM-0	UACACACCCCCCACCCCCCCCCCGCCCCCCCCCCCCCAAA	40	-47.0	-13.2
B-GMM-1	UCUCUGCUUACGCCAAAAUUCCCCCGGCCUAGCUUGGCUCGCUC	44	-35.9	21.9
B-GMM-2	ACGCCUACGCCAAAAGCCCCCCAGCCUGGCUUGGCGCGCAC	41	-29.9	-9.3
B-GMM-3	UGCGCGACCGCGCGCACAUUACGCGAAACUCCCCCCCGCC	40	-23.9	74.2
B-GMM-4	UGCGCGCCCAGCGCACAUUACGUAAAACUCCCCCCUACC	39	-23.3	229.1
B-GMM-5	UCGCCCUGCGCACAUAACUACGCAAAACUCCCCCCCGCCA	40	-26.8	-30.5
B-GMM-6	UCGCCUACGCAAAAAACUCCCCCUGCCUGUAUCACUCAC	39	-27.5	-28.5
B-GMM-7	UGCGCUACUCUACGCUAAACUCCCCCCAGCCUGGAAA	37	-26.4	-7.3
B-GMM-8	UGCGCGCCCGAGCGCACAUUACGCAAAAUCCCCCCUGCC	39	-24.5	190.7
B-GMM-9	UGCGAUACGCGAAACGCUCCCCCGCCUUCCUAG	33	-23.0	-7.3

Supplementary Table 3 continued from the previous page

ID	maximum probable sequences	length	log probability	relative activity
B-BO-0	UGCGCGAGCCGCGCCAUUACGCAACACUCGCCCCCUGCC	39	-22.0	5.6
B-BO-1	UGCGCGAGGCCGCGCCAUUACGUAACACUCGACCCCUACC	40	-21.8	3.2
B-BO-2	UGCGCGACCCGCGCCAUUACGCAACACCCCCCCUGCC	37	-21.6	79.4
B-BO-3	UGCGCGAGCCGCGCACAUUACGUAAAAAAUCGACCCCUACC	41	-24.3	13.5
B-BO-4	UGCGCGCCCAGCGCACAUUACGCAACACUCCCCCCUGCC	39	-23.8	231.0
B-BO-5	UGCGCGCCCGAGCGCACAUUACGUAAAACUCCCCCCUACC	40	-25.8	245.2
B-BO-6	UGCGCGACCGCGCGCACAUUACGCAACACCCCCCCUGCC	39	-23.4	93.7
B-BO-7	UGCGCGAGCAGCGCCAUUACGUAACACUCGCCCCCUACC	39	-23.3	45.2
B-BO-8	UGCGCGCCCGCGCGCACAUUACGCAACACUCCCCCCUGCC	40	-24.2	134.9
B-BO-9	UGCGCGCCCAGCGCACAUUACGUAAAAACCCCCCUACC	38	-23.7	151.6

Supplementary Section 3 Statistics of the Dataset

The statistics of the datasets are shown in Supplementary Table 4. The column names are as follows:

- ID : The ID named after the rule; {indicator of the dataset}-{round of the SELEX}.
- raw reads : The number of reads acquired in the sequencing procedure.
- no filter unique : The number of the unique sequences with no filtration.
- adapter match : The number of sequences that match the forward- and reverse-adapter.
- designed length : The number of sequences that match the adapters and also the length of the sequence matches the experimentally designed length.
- filtered unique : The number of unique sequences that passed both adapter filtering and design length filtering.
- > 1 : The number of filtered unique sequences that read more than once.
- $U(T)$: The unique ratio defined in the main paper. The ratio was calculated using filter-passed sequences.
- $\Delta U(T)$: The difference in unique ratio with the previous round.

Note that the data with the lowest $U(T)$, which holds $U(T) > 0.5$, were used.

Supplementary Table 4. Statistics of the datasets used in our research.

ID	raw reads	no filter unique	adapter match	designed length	filtered unique	> 1	$U(T)$	$\Delta U(T)$
A-0R	162003	159606	114717	93280	91899	1353	0.985	-
A-1R	91610	90225	72675	58555	57595	929	0.984	0.002
A-2R	45431	44829	36696	29296	28856	424	0.985	-0.001
A-3R	91441	90140	73054	58235	57349	857	0.985	0.000
**A-4R**	80864	65532	64503	51536	38513	3043	**0.747**	0.237
A-5R	108428	48575	86862	70785	20482	3760	0.289	0.458
A-6R	98237	25981	80801	67871	7750	2180	0.114	0.175
A-7R	49469	12565	40306	33101	3117	1118	0.094	0.020
A-8R	113137	26409	81177	67153	3312	1263	0.049	0.045
B-3R	146505	141174	102126	74454	72395	1874	0.972	-
**B-4R**	121185	85170	83310	58405	31358	4510	**0.537**	0.435
B-5R	116917	57404	83869	57955	13587	3375	0.234	0.302
B-6R	82488	37867	57827	34446	6064	1704	0.176	0.058

Supplementary Section 4 Sequence Logo Map

We created a map of sequence logos for the two sets of data acquired using the sequence logo creation method, as mentioned in Supplementary Section 1. This sequence logo is a visualization of the profile HMM at a point equally divided from -2.5 to 2.5 on each axis of the two-dimensional latent space. The sequence logo map for Dataset A is shown in Supplementary Figure 7, and for Dataset B is shown in Supplementary Figure 8.

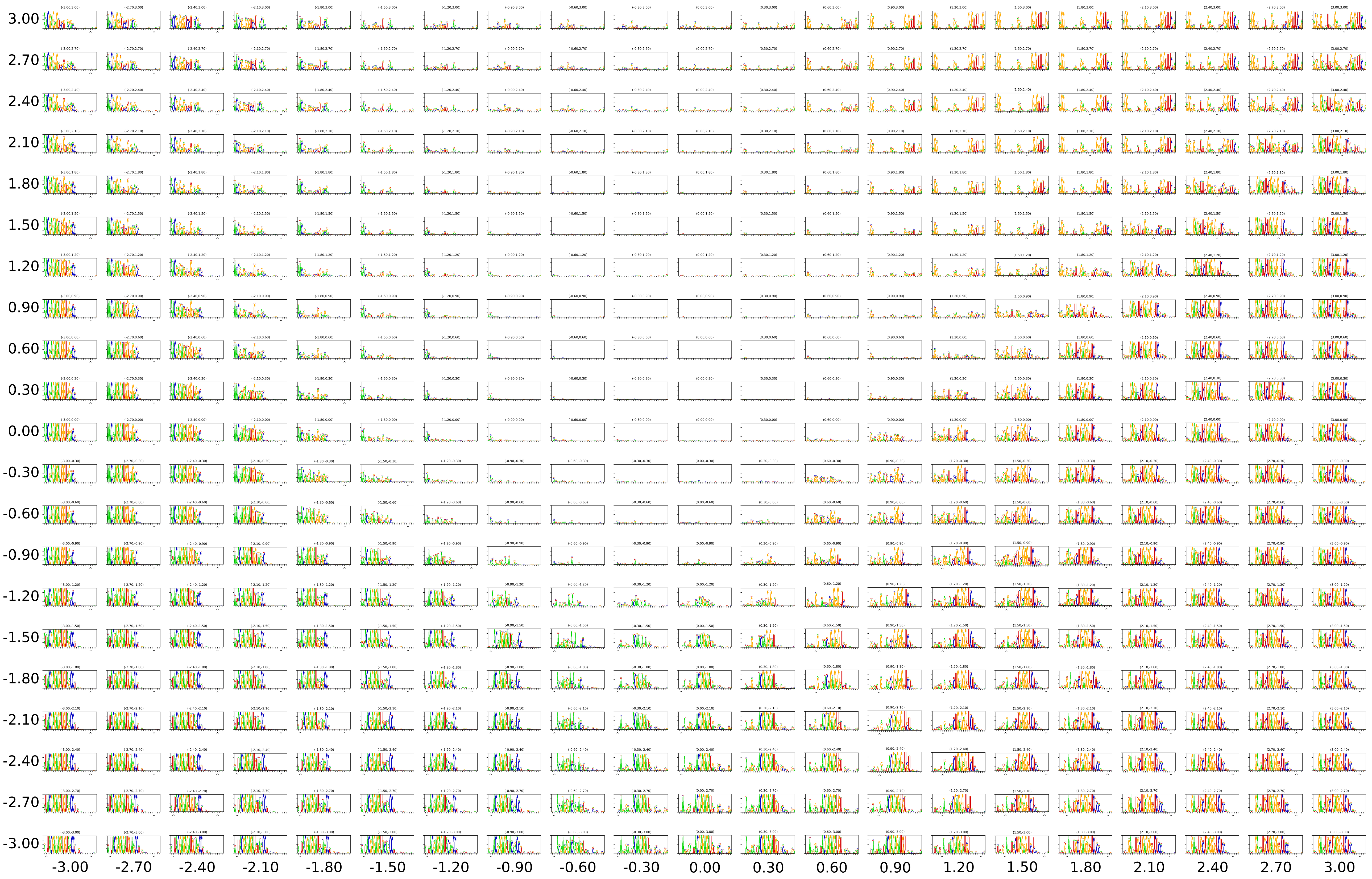

Supplementary Figure 7. The sequence logo map for Dataset A. The continuous motif indicated that the sequences in the data had the preference for specific subsequences. The result was partly consistent with our previous research [5] that implied `AGAGAUGGUA` was the truncated motif.

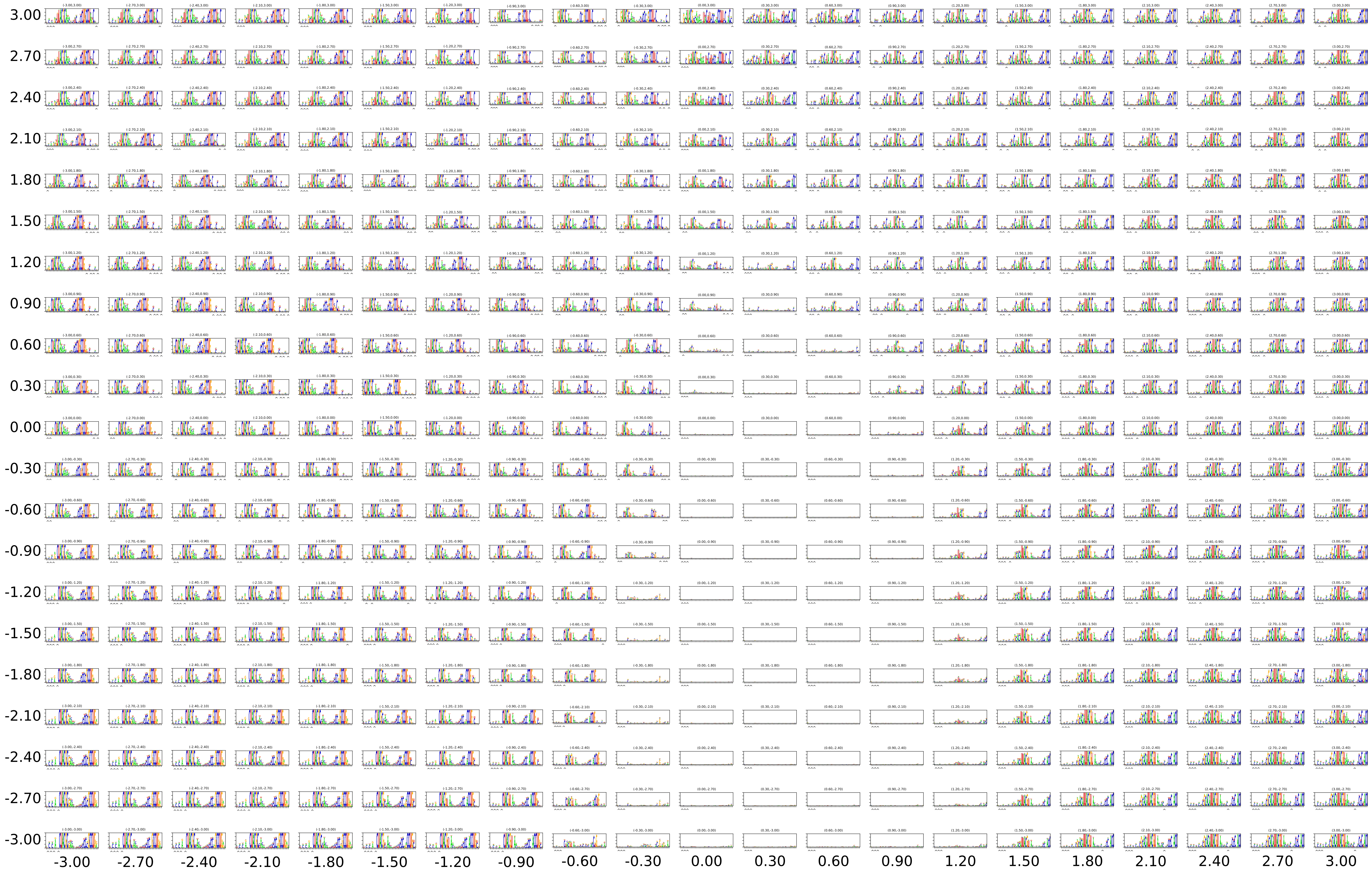

Supplementary Figure 8. The sequence logo map for Dataset B. The logos indicate that the Profile HMM at the center of the latent space had no preference to emit sequences while the outer surrounding points had captured split motifs.

Supplementary Section 5 Structure of RaptGen

The structure of RaptGen is shown in Supplementary Figure 9. The RaptGen consists of a convolutional neural network (CNN)-based encoder and a profile HMM for decoder distribution. We further tried recurrent neural networks with long short-term memory (LSTM) and CNN-LSTM, a network of CNN followed by LSTM. The CNN utilized skip-connection [6], which enables deeper layers to learn appropriately. The models implemented in this study are available at `https://github.com/hmdlab/raptgen`.

Supplementary Section 6 Encoders

Supplementary Section 6.1 Convolutional neural network

A CNN captures sequential motifs that are aligned in certain positions. The encoder CNN is a network that first embeds each character into a 32-dimensional vector. Then, the six layers of the skip-connection layer capture the interactions. Finally, the max-pooling layer outputs the resulting feature vector. The skip-connection layer is a combination of a convolutional layer, batch normalization [7], and leaky rectified linear unit (leaky ReLU) [8]. The structure of the skip-connection layer is

$$\begin{aligned}
\mathbf{x}_1 &= \mathrm{Conv}_1(\sigma(\mathrm{BN}(\mathbf{x}_{\mathrm{in}}))) \\
\mathbf{x}_2 &= \mathrm{Conv}_2(\sigma(\mathrm{BN}(\mathbf{x}_1))) \\
\mathbf{x}_3 &= \mathrm{Conv}_3(\sigma(\mathrm{BN}(\mathbf{x}_2))) \\
\mathbf{x}_{out} &= \sigma(\mathbf{x}_{\mathrm{in}} + \mathbf{x}_3)
\end{aligned}$$

where $\mathbf{x}_{\mathrm{in}}$ is the input vector, $\mathrm{BN}(\cdot)$ is the batch normalization layer, and $\sigma(\cdot)$ is the leaky ReLU layer. Convolutional layer Conv_1, Conv_2, and Conv_3 transforms vector dimensions from 32 to 64, 64 to 64, and 64 to 32, respectively. All convolutional layers had the same kernel size of 7 and a zero-padding length of 3 on both edges. Finally, max-pooling, taking the maximum value along the sequence, was performed, resulting in a 32-dimensional feature vector. An encoder CNN was used in the RaptGen architecture.

Supplementary Section 6.2 Recurrent neural network with long short-term memory

A recurrent neural network can consider the context of the input sequence. LSTM is an artifact that can handle the gradient vanishing problem, the hardness to learn long sequential data [9]. We used bidirectional LSTM for encoding sequences. The sequence was first embedded into a 32-dimensional vector, similar to the CNN encoder, and then it was calculated through a 16-dimensional hidden vector in each direction. The final hidden vector for each direction was concatenated into a 32-dimensional vector, and this was used for the feature vector.

Supplementary Section 6.3 CNN-LSTM

The convolutional layer and recurrent layer are used in combination to consider both fixed-length and long-distance interactions. The CNN was almost the same as described in the previous section, with the difference that the final max-pooling was removed. LSTM treated this feature vector as sequence embedding as described in Supplementary Section 6.2.

Supplementary Section 7 Decoders

Supplementary Section 7.1 Multi categorical model

The multi-categorical model gives the probability to each position of the fixed-length sequence. The output of the model is

$$\begin{aligned}\mathbf{x}_1 &= \sigma(\mathrm{BN}(\mathrm{FC}_{\mathrm{D},32}(\mathbf{z})))\\ \mathbf{x}_2 &= \sigma(\mathrm{BN}(\mathrm{FC}_{32,64}(\mathbf{x}_1)))\\ \mathbf{x}_3 &= \sigma(\mathrm{BN}(\mathrm{FC}_{64,32}(\mathbf{x}_2)))\\ \mathbf{x}_4 &= \mathrm{FC}_{32,32\times L}(\mathbf{x}_1+\mathbf{x}_3)\end{aligned}$$

Where $\mathbf{z}$ is the sampled vector and $\mathrm{FC}_{J,K}$ is a fully connected layer, which is defined as $\mathrm{FC}_{J,K}(\mathbf{x}) = W_{\mathrm{J,K}}^{\mathrm{T}}\mathbf{x}$ with the learnable parameter $W_{\mathrm{J,K}} \in \mathcal{R}^{\mathrm{J}\times\mathrm{K}}$. To interpret the interactions of each other, the embedding parameter is calculated using the transposed convolution function [10], which is generally the opposite of a convolutional function. The final output $\mathbf{x}_{\mathrm{out}}$ is

$$\begin{aligned}\mathbf{x}_5 &= \mathrm{Trans\_Conv}_{32,32}(\sigma(\mathrm{BN}(\mathbf{x}_4)))\\ \mathbf{x}_6 &= \mathrm{Trans\_Conv}_{32,32}(\sigma(\mathrm{BN}(\mathbf{x}_5)))\\ \mathbf{x}_{\mathrm{out}} &= \sigma(\mathrm{Trans\_Conv}_{32,32}(\sigma(\mathrm{BN}(\mathbf{x}_6))))\end{aligned}$$

where $\mathrm{Trans\_Conv}_{\mathrm{J,K}}(\cdot)$ is the transposed convolutional function with the trainable parameters of the input dimension J and output dimension K. All transposed convolutional layers have the same kernel size of 7 and the same padding length of 3.

Supplementary Section 7.2 Autoregressive model

To run the autoregressive model, we used a gated recurrent unit (GRU), which is a simplified version of LSTM [11]. The GRU is calculated as follows:

$$\begin{aligned}z_t &= \sigma_g\left(W_z x_t + U_z h_{t-1} + b_z\right)\\ r_t &= \sigma_g\left(W_r x_t + U_r h_{t-1} + b_r\right)\\ h_t &= (1-z_t)\circ h_{t-1} + z_t \circ \sigma_h\left(W_h x_t + U_h\left(r_t \circ h_{t-1}\right) + b_h\right)\end{aligned}$$

where t is time, x_t is the input vector, h_t is the output vector, z_t is the update gate vector, r_t is the initializing gate vector, and W, U, and b are parameter vectors. The probability of output sequence $p(\mathbf{x}) = \prod_{i=1}^{L} p(x_i \mid x_{0:i-1}, \mathbf{z})$ is calculated by

$$\begin{aligned}\mathbf{x}_1 &= \sigma(\mathrm{BN}(\mathrm{FC}_{\mathrm{D},32}(\mathbf{z})))\\ \mathbf{x}_2 &= \sigma(\mathrm{BN}(\mathrm{FC}_{32,64}(\mathbf{x}_1)))\\ \mathbf{x}_3 &= \sigma(\mathrm{BN}(\mathrm{FC}_{64,32}(\mathbf{x}_2)))\\ \mathbf{x}_4 &= \mathrm{GRU}(\mathbf{x}_{\mathrm{in}}, \mathbf{x}_1+\mathbf{x}_3)\\ \mathbf{x}_{\mathrm{out}} &= \sigma(\mathrm{FC}_{32,32}(\mathbf{x}_4))\end{aligned}$$

where $\mathrm{GRU}(\mathbf{x}, h_0)$ is defined as a GRU function with input vector $\mathbf{x}$ of length L and initial hidden vector h_0, which outputs h_L.

Supplementary Section 7.3 Profile hidden Markov model

The profile HMM is described in Supplementary Figure 9b. The embedded sequence is a D-dimensional vector, which is transformed into 32 dimensions by a fully connected (FC) layer. After the vector is rectified by leaky ReLU, the vector is transformed into a certain shape to fit the parameters of Profile HMM. For each parameter, FC, leaky ReLU, FC, and reshape procedure is performed.

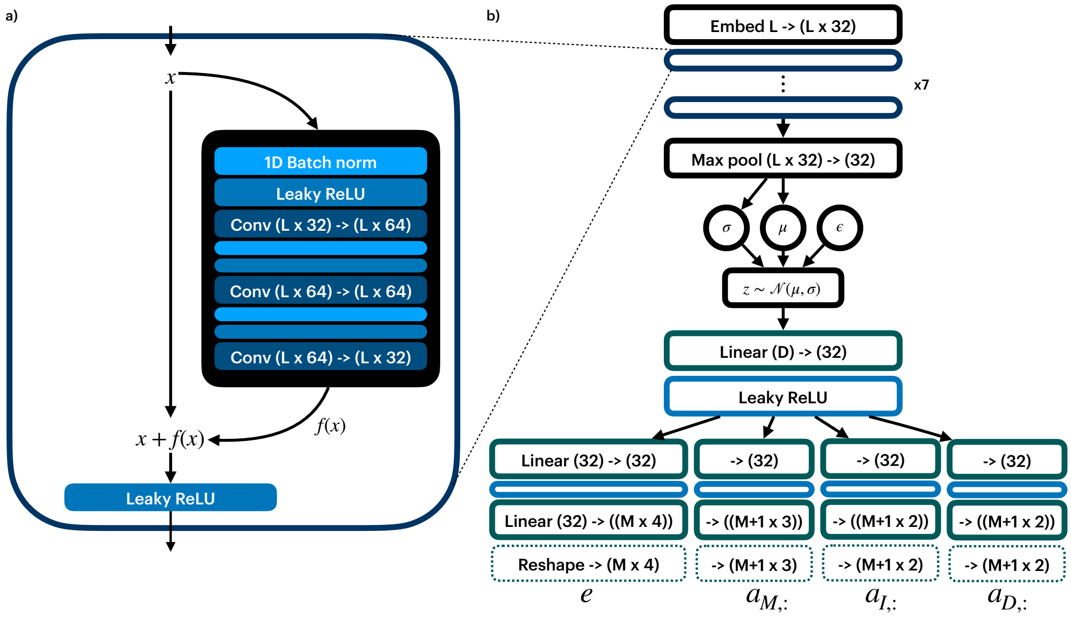

Supplementary Figure 9. (a) The skip-connection layer. The input feature vector is first passed through 64 hidden layers, then through 32 layers, and added to the original vector. It then passes through the Leaky ReLU normalization layer and produces output. (b) Overall architecture of RaptGen. The input sequence is initially embedded into 32 feature vector and goes through skip-connection layers. After the latent mean and log-variance are calculated with the fully connected layer, which is written as "Linear," the latent variable is sampled and calculated to fit the profile HMM parameter shapes.

Supplementary Section 8 The Embeddings of Different Encoder and Decoder Combinations

The embeddings are shown in Supplementary Figure 10, where the row and column indicate encoder and decoder, respectively. The embedding of the multi-categorical probabilistic model tends to place sequences near the same motif; however, the nearest surrounding sequence is not from the same motifs. Although the autoregressive model has a lower loss, it tends to have an unsplit latent space.

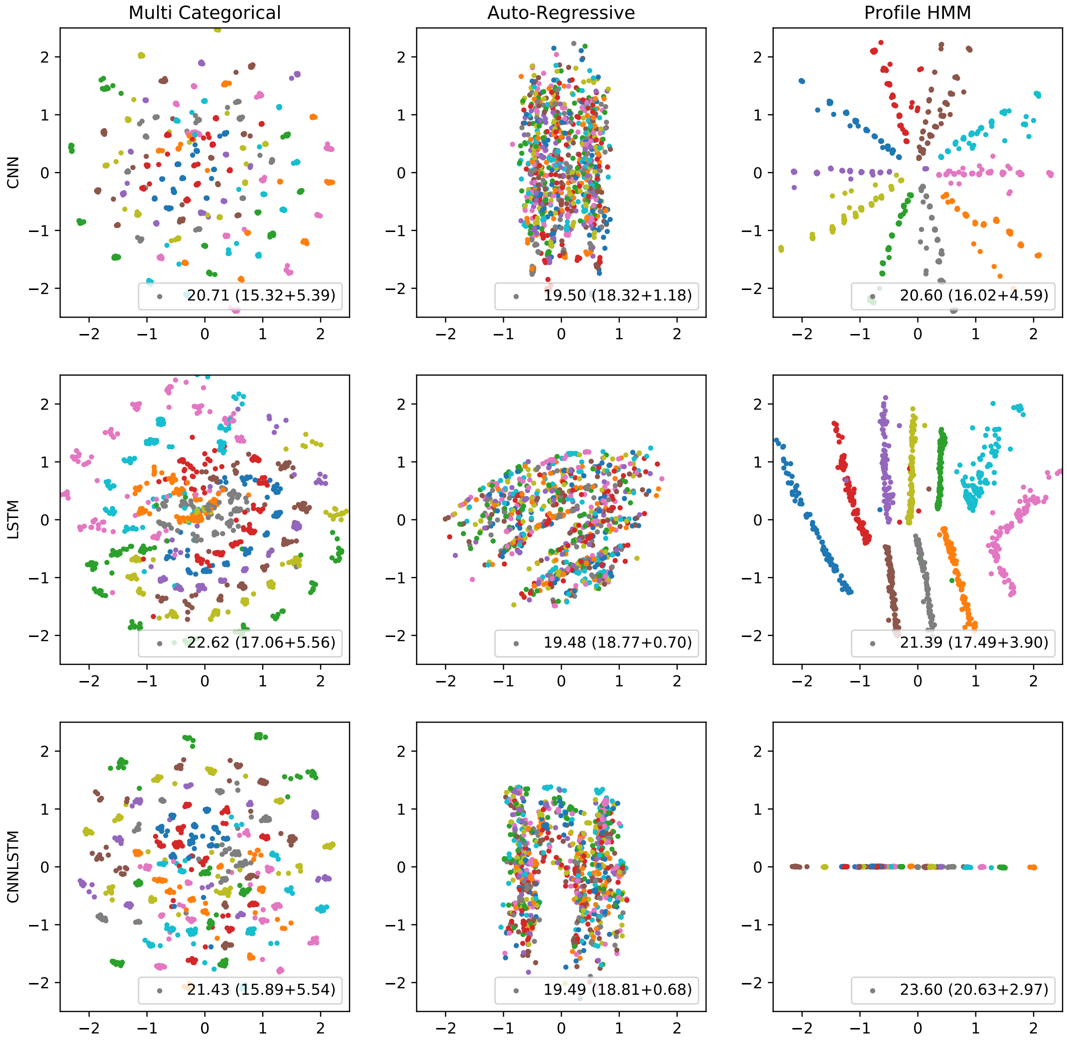

Supplementary Figure 10. Embeddings of different encoders (row) and decoders (column). The minimum loss is written at the bottom-right corner. The reconstruction error is to the left, and the regularization error is to the right in the braces. The color represents the motif that each sequence contains.

Supplementary Section 9 Application of RaptGen to SELEX for transcription factors

Jolma and colleagues evaluated the binding specificities of transcription factors using SELEX data [12]. In addition, CNN-based research was conducted to classify randomly shuffled sequences and determine the motifs in a dataset [13]. We selected five transcription factors that were presented in these studies. We applied ten GMM distributions in this experiment and identified motifs similar to those from Jolma *et al.* Similarity was based on the edit distance of the most probable sequence from each motif. RaptGen was able to produce sequence motifs consistent with these studies (Supplementary Table 5). Thus, we could search for sequence motifs obtained by running a CNN. A future study could be performed to search for an appropriate number of distributions using model selection methods such as Akaike Information Criteria (AIC) [14]. Supplementary Table 6 shows learned embedding spaces and the sequence logo of the GMM center trained on 10 components. Although the embeddings did not clearly split into 10 areas, the Profile HMM logo was consistent with previously determined motifs. Logo images of previous research were downloaded from the CisBP database [15]. The motif learned by Deepbind with the top three weights is also shown.

Supplementary Table 5. The motif similarity with previous methods. RaptGen motif was generated as described in Supplementary Supplementary Section 1. The Motif of RaptGen is based on the emission probabilities, motifs of Jolma et al. are based on position frequency matrix (PFM) of the multinomial method [16]. The motifs of Alipanahi et al. were based on the PFM, which activated specific motif scanners of the DeepBind model [13], which were derived from deepbind web service `http://tools.genes.toronto.edu/deepbind/`.

Target	RaptGen	Jolma *et al.*	Alipanahi *et al.*
CTCF	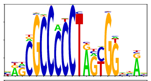	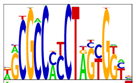	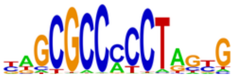
NR4A2	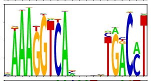	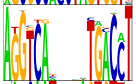	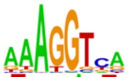
SOX10	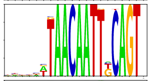	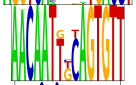	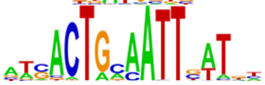
EBF1	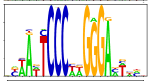	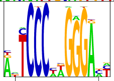	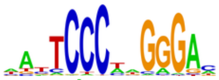
POU2F2	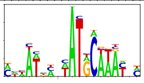	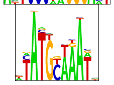	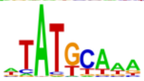

Supplementary Table 6. Sequences that were taken from the SELEX experiment in a previous study [12] are shown. Position interdependent motifs (CTCF, NR4A2), secondary motifs(Sox10, Pou2f2), and motif suggesting potential co-factors (EBF1) are presented.

target	embeddings	idx	seq logo	idx	seq logo	jolma et al.	Alipanahi et al.
CTCF	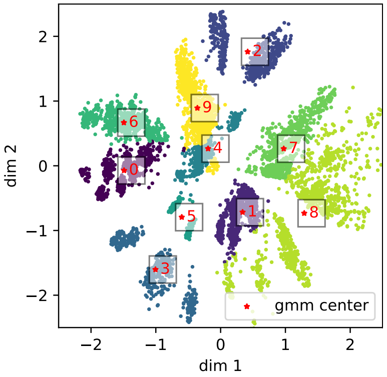	0<br>1<br>2<br>3<br>4	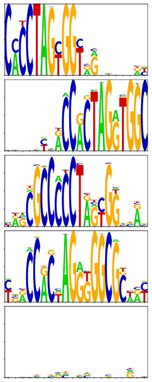	5<br>6<br>7<br>8<br>9	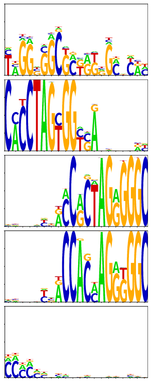	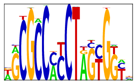	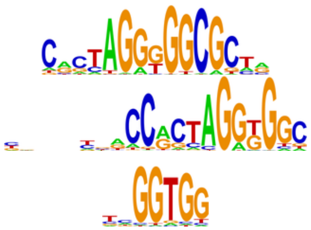
NR4A2	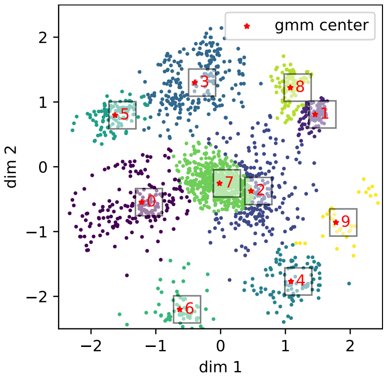	0<br>1<br>2<br>3<br>4	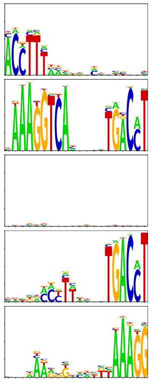	5<br>6<br>7<br>8<br>9	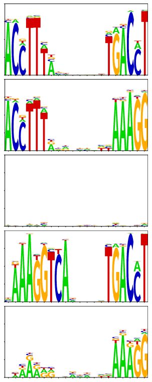	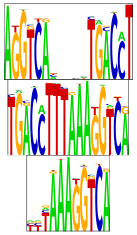	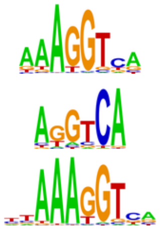
SOX10	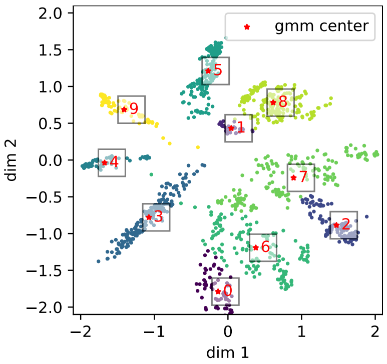	0<br>1<br>2<br>3<br>4	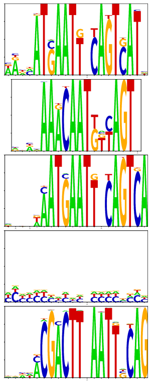	5<br>6<br>7<br>8<br>9	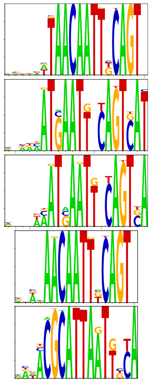	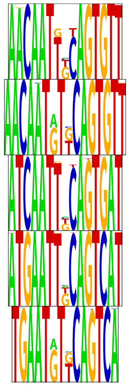	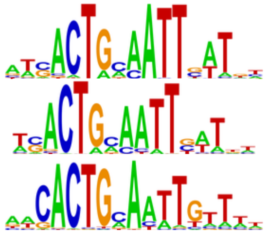

Supplementary Table 6 continued from the previous page

target	embeddings	idx	seq logo	idx	seq logo	Jolma et al.	Alipanahi et al.
POU2F2	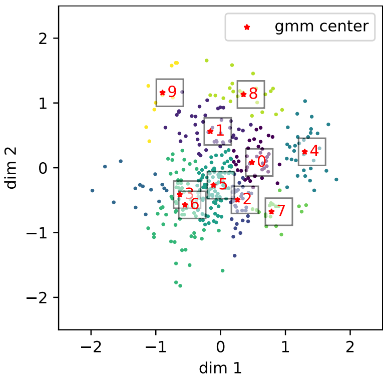	0	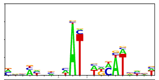	5	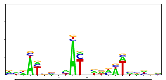	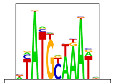	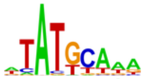
		1	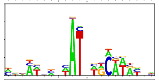	6	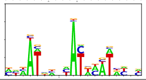	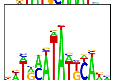	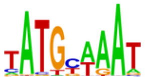
		2	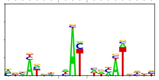	7	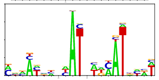		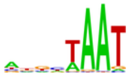
		3	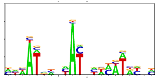	8	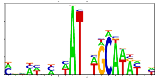		
		4	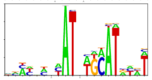	9	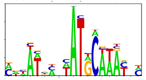		
EBF1	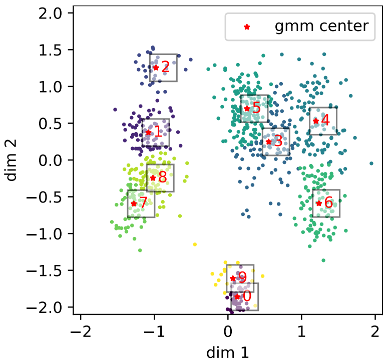	0	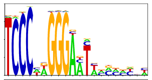	5	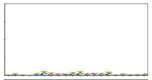	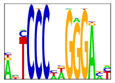	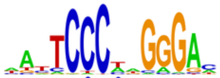
		1	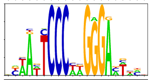	6	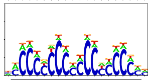		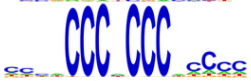
		2	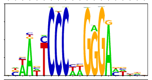	7	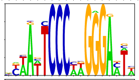		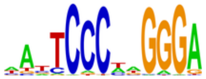
		3	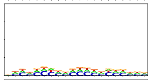	8	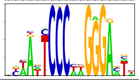		
		4	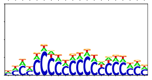	9	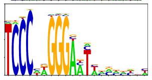		

References

1. M. Hamada, H. Kiryu, K. Sato, T. Mituyama, and K. Asai. Prediction of RNA secondary structure using generalized centroid estimators. *Bioinformatics*, 25(4):465–473, Feb 2009.

2. R. Lorenz, S. H. Bernhart, C. Höner Zu Siederdissen, H. Tafer, C. Flamm, P. F. Stadler, and I. L. Hofacker. ViennaRNA Package 2.0. *Algorithms Mol Biol*, 6:26, Nov 2011.

3. Thomas D Schneider and R Michael Stephens. Sequence logos: a new way to display consensus sequences. *Nucleic acids research*, 18(20):6097–6100, 1990.

4. Gavin E Crooks, Gary Hon, John-Marc Chandonia, and Steven E Brenner. Weblogo: a sequence logo generator. *Genome research*, 14(6):1188–1190, 2004.

5. Ryoga Ishida, Tatsuo Adachi, Aya Yokota, Hidehito Yoshihara, Kazuteru Aoki, Yoshikazu Nakamura, and Michiaki Hamada. Raptranker: in silico rna aptamer selection from ht-selex experiment based on local sequence and structure information. *Nucleic acids research*, 48(14):e82–e82, 2020.

6. Kaiming He, Xiangyu Zhang, Shaoqing Ren, and Jian Sun. Deep residual learning for image recognition. In *Proceedings of the IEEE conference on computer vision and pattern recognition*, pages 770–778, 2016.

7. Sergey Ioffe and Christian Szegedy. Batch normalization: Accelerating deep network training by reducing internal covariate shift. *arXiv preprint arXiv:1502.03167*, 2015.

8. Andrew L Maas, Awni Y Hannun, and Andrew Y Ng. Rectifier nonlinearities improve neural network acoustic models. In *Proc. icml*, volume 30, page 3, 2013.

9. Sepp Hochreiter and Jürgen Schmidhuber. Long short-term memory. *Neural computation*, 9(8):1735–1780, 1997.

10. Vincent Dumoulin and Francesco Visin. A guide to convolution arithmetic for deep learning. *arXiv preprint arXiv:1603.07285*, 2016.

11. Kyunghyun Cho, Bart Van Merriënboer, Caglar Gulcehre, Dzmitry Bahdanau, Fethi Bougares, Holger Schwenk, and Yoshua Bengio. Learning phrase representations using rnn encoder-decoder for statistical machine translation. *arXiv preprint arXiv:1406.1078*, 2014.

12. Arttu Jolma, Jian Yan, Thomas Whitington, Jarkko Toivonen, Kazuhiro R Nitta, Pasi Rastas, Ekaterina Morgunova, Martin Enge, Mikko Taipale, Gonghong Wei, et al. Dna-binding specificities of human transcription factors. *Cell*, 152(1-2):327–339, 2013.

13. Babak Alipanahi, Andrew Delong, Matthew T Weirauch, and Brendan J Frey. Predicting the sequence specificities of dna-and rna-binding proteins by deep learning. *Nature biotechnology*, 33(8):831–838, 2015.

14. Hirotugu Akaike. Information theory as an extension of the maximum likelihood principle. In *Second International Symposium on Information Theory. Akademiai Kiado, Budapest*, pages 276–281. B.N. Petrov, F. Csaki (Eds.), 1973.

15. Matthew T Weirauch, Ally Yang, Mihai Albu, Atina G Cote, Alejandro Montenegro-Montero, Philipp Drewe, Hamed S Najafabadi, Samuel A Lambert, Ishminder Mann, Kate Cook, et al. Determination and inference of eukaryotic transcription factor sequence specificity. *Cell*, 158(6):1431–1443, 2014.

16. Arttu Jolma, Teemu Kivioja, Jarkko Toivonen, Lu Cheng, Gonghong Wei, Martin Enge, Mikko Taipale, Juan M Vaquerizas, Jian Yan, Mikko J Sillanpää, et al. Multiplexed massively parallel selex for characterization of human transcription factor binding specificities. *Genome research*, 20(6):861–873, 2010.
