# Supplementary material for: Generative aptamer discovery using RaptGen
Source: Nat Comput Sci. 2022 Jun 2;2(6):378–86. doi: 10.1038/s43588-022-00249-6 (PMC10766510; doi:10.1038/s43588-022-00249-6)
Supplement: Supplementary file 2 — Reporting Summary [file 43588_2022_249_MOESM2_ESM.pdf]

nature research

Corresponding author(s): Michiaki Hamada

Last updated by author(s): Apr 19, 2022

Reporting Summary

Nature Research wishes to improve the reproducibility of the work that we publish. This form provides structure for consistency and transparency in reporting. For further information on Nature Research policies, see our Editorial Policies and the Editorial Policy Checklist.

Statistics

For all statistical analyses, confirm that the following items are present in the figure legend, table legend, main text, or Methods section.

n/a	Confirmed	
☐	☒	The exact sample size (*n*) for each experimental group/condition, given as a discrete number and unit of measurement
☒	☐	A statement on whether measurements were taken from distinct samples or whether the same sample was measured repeatedly
☒	☐	The statistical test(s) used AND whether they are one- or two-sided<br>*Only common tests should be described solely by name; describe more complex techniques in the Methods section.*
☒	☐	A description of all covariates tested
☒	☐	A description of any assumptions or corrections, such as tests of normality and adjustment for multiple comparisons
☒	☐	A full description of the statistical parameters including central tendency (e.g. means) or other basic estimates (e.g. regression coefficient) AND variation (e.g. standard deviation) or associated estimates of uncertainty (e.g. confidence intervals)
☒	☐	For null hypothesis testing, the test statistic (e.g. *F*, *t*, *r*) with confidence intervals, effect sizes, degrees of freedom and *P* value noted<br>*Give P values as exact values whenever suitable.*
☒	☐	For Bayesian analysis, information on the choice of priors and Markov chain Monte Carlo settings
☒	☐	For hierarchical and complex designs, identification of the appropriate level for tests and full reporting of outcomes
☒	☐	Estimates of effect sizes (e.g. Cohen's *d*, Pearson's *r*), indicating how they were calculated

Our web collection on statistics for biologists contains articles on many of the points above.

Software and code

Policy information about availability of computer code

Data collection	Biacore T200 control software version 2.0<br>Biacore T200 evaluation software
Data analysis	CentroidFold (Web version: http://rtools.cbrc.jp/centroidfold/)<br>RNAfold 2.5.0<br>click 7.1.2<br>scikit-learn 0.23.2<br>tqdm 4.41.1<br>matplotlib 3.1.2<br>pandas 1.1.5<br>numpy 1.18.1<br>torch 1.5.0<br>torchvision 0.6.0<br>svgwrite 1.4<br>scipy 1.4.0<br>GPy 1.9.9<br>GPyOpt 1.2.6<br>CairoSVG 2.5.1

For manuscripts utilizing custom algorithms or software that are central to the research but not yet described in published literature, software must be made available to editors and reviewers. We strongly encourage code deposition in a community repository (e.g. GitHub). See the Nature Research guidelines for submitting code & software for further information.

Data

Policy information about availability of data

All manuscripts must include a data availability statement. This statement should provide the following information, where applicable:

- Accession codes, unique identifiers, or web links for publicly available datasets
- A list of figures that have associated raw data
- A description of any restrictions on data availability

Source data for Figs. 2–6 are provided with this paper. HT-SELEX sequences are available as DRA009383 (for Dataset A) and DRA009384 (Dataset B) in DDBJ.

Field-specific reporting

Please select the one below that is the best fit for your research. If you are not sure, read the appropriate sections before making your selection.

☒ Life sciences ☐ Behavioural & social sciences ☐ Ecological, evolutionary & environmental sciences

For a reference copy of the document with all sections, see nature.com/documents/nr-reporting-summary-flat.pdf

Life sciences study design

All studies must disclose on these points even when the disclosure is negative.

Sample size	No sample size calculation were performed. More than one active sample is enough to show the aptamer-generation-capability of the software. Therefore, no statistical test was introduced. We chose ten aptamers for each generation setting because of the throughput of the surface plasmon resonance machine.
Data exclusions	No data were excluded from the analyses.
Replication	We ran surface plasmon resonance analyses on 50 and 40 different candidate aptamers from dataset A and B, respectively. For each binding measurement, we performed the resonance unit measurement once for each candidate aptamer. To validate the binding activity of predicated aptamers, a positive control aptamer was measured every five or ten cycles for dataset A or B, respectively. Relative activity was obtained as a relative value to the control aptamer.We confirmed all control aptamer evaluation was successfully replicated.
Randomization	This is not relevant to our study. Aptamer generation was not compared to control groups
Blinding	This is not relevant to our study. Aptamer selection and affinity measurements were done with a machine, no human judgment was involved.

Reporting for specific materials, systems and methods

We require information from authors about some types of materials, experimental systems and methods used in many studies. Here, indicate whether each material, system or method listed is relevant to your study. If you are not sure if a list item applies to your research, read the appropriate section before selecting a response.

Materials & experimental systems

n/a	Involved in the study
☒	☐ Antibodies
☒	☐ Eukaryotic cell lines
☒	☐ Palaeontology and archaeology
☒	☐ Animals and other organisms
☒	☐ Human research participants
☒	☐ Clinical data
☒	☐ Dual use research of concern

Methods

n/a	Involved in the study
☒	☐ ChIP-seq
☒	☐ Flow cytometry
☒	☐ MRI-based neuroimaging
