# Supplementary material for: Generative aptamer discovery using RaptGen
Source: Nat Comput Sci. 2022 Jun 2;2(6):378–86. doi: 10.1038/s43588-022-00249-6 (PMC10766510; doi:10.1038/s43588-022-00249-6)
Supplement: Supplementary file 3 — The detailed results for secondary structure analyses. [file 43588_2022_249_MOESM3_ESM.pdf]

ID to discriminate every tested sequence	A: Dataset A,<br>B: Dataset B	Which method used to select;<br>BO: Bayesian Optimization,<br>GMM: Gaussian Mixture Model	The length of a profile HMM model	The maximum probable pattern generated from profile HMM model. The asterisk indicates insertion.	The maximum probable sequence derived from "max_model"	The log probability of a sequence generated from the profile HMM	Normalized binding affinity of the proposed sequence	The **x** position where the profile HMM model was taken	The **y** position where the profile hmm model was taken	The length of the derived sequence	Base pairing probability matrix (upper-right); Secondary structure (lower-left)	Secondary structure calculated by CentroidFold[^1]<br>[^1]: https://doi.org/10.1093/bioinformatics/btn601	Structual profiles calculated by CapR[^2], where the horizontal axis show positions of sequence<br>[^2]: https://doi.org/10.1186/gb-2014-15-1-r16	Secondary structure calculated by RNAfold[^3]<br>[^3]: https://doi.org/10.1186/1748-7188-6-26
**id**	**data**	**method**	**target_len**	**max_model**	**max_seq**	**log_proba**	**normalized**	**pos_x**	**pos_y**	**seq_len**	**base_paring_probabilities**	**secondary_structure**	**CapR**	**secondary_structure(RNAfold)**
**A-PC**	A				GCGGAGAUUCUGAGGGUUCUCCUGUUCCAG		100				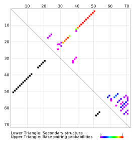	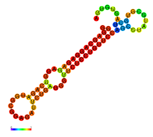	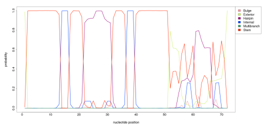	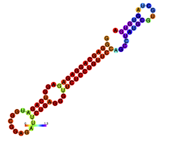
**A-BO-3**	A	BO	30	GUAGAGAUUCUGAGGGUUCUCCUGUU*CUA	GUAGAGAUUCUGAGGGUUCUCCUGUUGCUA	-14.35	100.6	1.26	-0.43	30	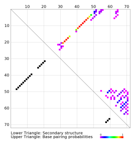	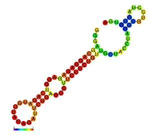	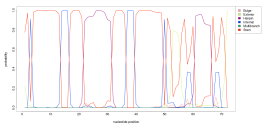	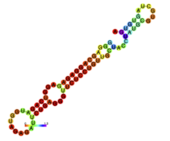
**A-BO-1**	A	BO	30	GUUGAGAUUCUGAGGGUUCUCCUGUU*CCC	GUUGAGAUUCUGAGGGUUCUCCUGUUGCCC	-14.42	101.2	1.69	-0.55	30	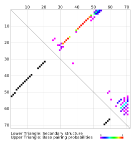	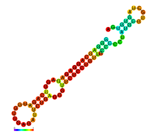	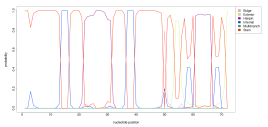	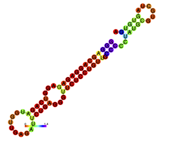
**A-BO-0**	A	BO	30	GUAGAGAUUCUGAGGGUUCUCCUGUU*ACC	GUAGAGAUUCUGAGGGUUCUCCUGUUGACC	-14.42	102.5	1.55	-0.20	30	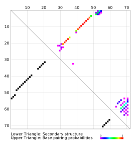	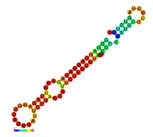	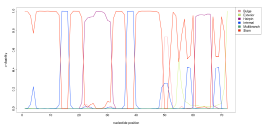	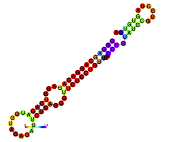
**A-BO2-8**	A	BO	30	GGUAGAGAUUCUGAGGGUUCUCCUGUUCUA	GGUAGAGAUUCUGAGGGUUCUCCUGUUCUA	-16.12	105.8	0.90	-0.44	30	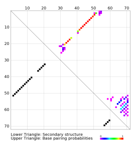	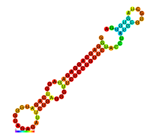	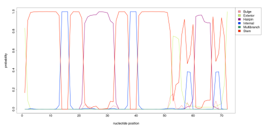	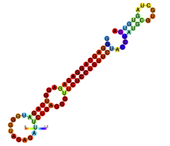
**A-GMM-1**	A	GMM	30	GUAGAGAUUCUGAGGGUUCUCCUGCUAUA	GUAGAGAUUCUGAGGGUUCUCCUGCUAUA	-13.33	107.1	1.36	0.22	29	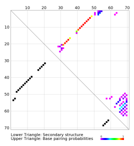	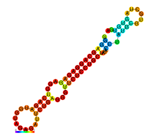	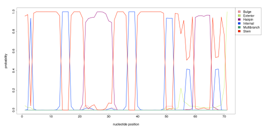	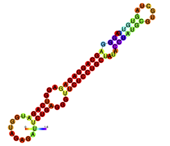
**A-BO2-7**	A	BO	30	GUAGAGAUUCUGAGGGUUCUCCUGUU*CCA	GUAGAGAUUCUGAGGGUUCUCCUGUUGCCA	-14.43	116.2	1.51	-0.63	30	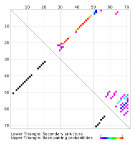	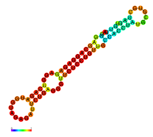	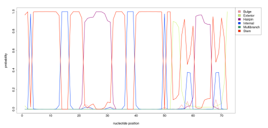	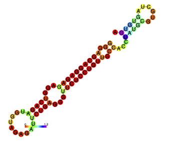
**A-BO2-2**	A	BO	30	GUAGAGAUUCUGAGGGUUCUCCUGUUACA	GUAGAGAUUCUGAGGGUUCUCCUGUUACA	-13.88	119.8	1.29	-0.08	29	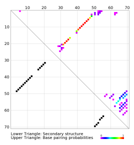	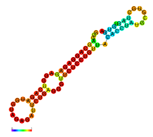	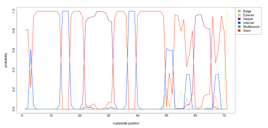	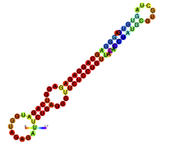

ID to discriminate every tested sequence	A: Dataset A, B: Dataset B	Which method used to select; BO: Bayesian Optimization, GMM: Gaussian Mixture Model	The length of a profile HMM model	The maximum probable pattern generated from profile HMM model. The asterisk indicates insertion.	The maximum probable sequence derived from "max_model"	The log probability of a sequence generated from the profile HMM	Normalized binding affinity of the proposed sequence	The **x** position where the profile HMM model was taken	The **y** position where the profile hmm model was taken	The length of the derived sequence	Base pairing probability matrix (upper-right); Secondary structure (lower-left)	Secondary structure calculated by CentroidFold[^1] [^1]: https://doi.org/10.1093/bioinformatics/btn601	Structual profiles calculated by CapR[^2], where the horizontal axis show positions of sequence [^2]: https://doi.org/10.1186/gb-2014-15-1-r16	Secondary structure calculated by RNAfold[^3] [^3]: https://doi.org/10.1186/1748-7188-6-26
**id**	**data**	**method**	**target_len**	**max_model**	**max_seq**	**log_proba**	**normalized**	**pos_x**	**pos_y**	**seq_len**	**base_paring_probabilities**	**secondary_structure**	**CapR**	**secondary_structure(RNAfold)**
**B-PC**	B				GCUGUGUCUACGUCCGGAUUGGGGACCUGCACGGCCCAUG		100				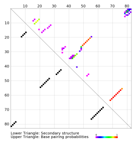	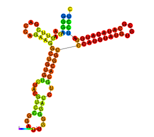	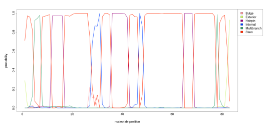	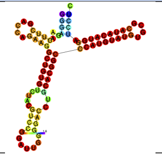
**B-BO-8**	B	BO	40	U*C*C*CCC*CGCGCACAUUACGCAACACUCCCCCCUGCC	UGCGCGCCCGCGCGCACAUUACGCAACACUCCCCCCUGCC	-24.20	134.9	1.55	-0.53	40	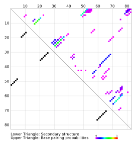	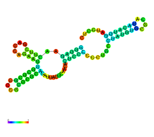	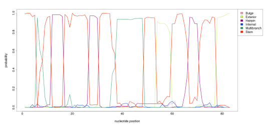	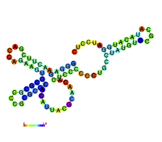
**B-BO-9**	B	BO	40	U*C*C*CCCAGCGCACAUUACGUAAAAACCCCCCUACC	UGCGCGCCCAGCGCACAUUACGUAAAAACCCCCCUACC	-23.65	151.6	0.99	-1.17	38	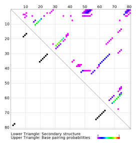	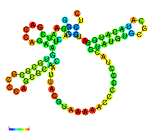	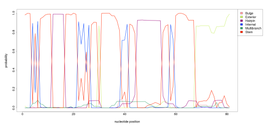	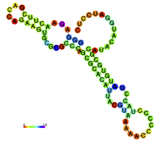
**B-GMM-9**	B	GMM	40	U*C*C*CCC*AGCGCACAUUACGCAAAAUCCCCCCUGCC	UGCGCGCCCGAGCGCACAUUACGCAAAAUCCCCCCUGCC	-24.47	190.7	1.24	0.07	39	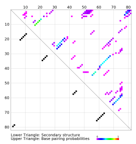	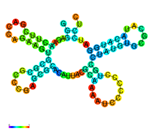	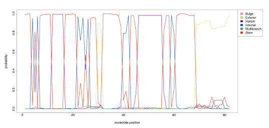	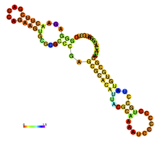
**B-GMM-4**	B	GMM	40	U*C*C*CCCAGCGCACAUUACGUAAAACUCCCCCCUACC	UGCGCGCCCAGCGCACAUUACGUAAAACUCCCCCCUACC	-23.33	229.1	1.10	-0.99	39	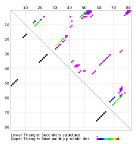	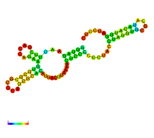	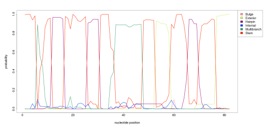	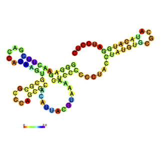
**B-BO-4**	B	BO	40	U*C*C*CCCAGCGCACAUUACGCAACACUCCCCCCUGCC	UGCGCGCCCAGCGCACAUUACGCAACACUCCCCCCUGCC	-23.80	231.0	1.53	-0.81	39	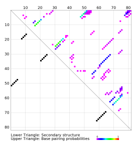	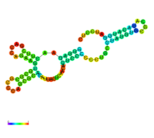	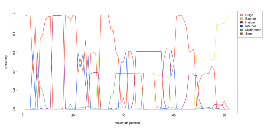	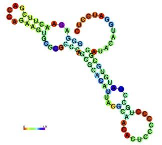
**B-BO-5**	B	BO	40	U*C*C*CCC*AGCGCACAUUACGUAAAACUCCCCCCUACC	UGCGCGCCCGAGCGCACAUUACGUAAAACUCCCCCCUACC	-25.79	245.2	1.23	-0.68	40	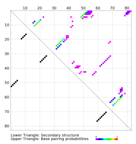	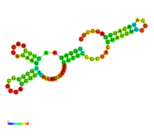	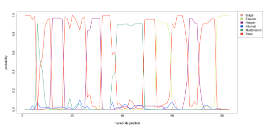	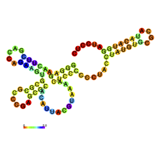
